# Mechanosensitive lncRNA H19 promotes chondrocyte autophagy, but not pyroptosis, by targeting miR-148a in post-traumatic osteoarthritis

**DOI:** 10.1016/j.ncrna.2024.07.005

**Published:** 2024-07-31

**Authors:** Xuchang Zhou, Hong Cao, Tao Liao, Weizhong Hua, Ruobing Zhao, Dongxue Wang, Huili Deng, Yajing Yang, ShengYao Liu, Guoxin Ni

**Affiliations:** a School of Sport Medicine and Rehabilitation, Beijing Sport University, Beijing, 100084, China; b School of Kinesiology, Shanghai University of Sport, Shanghai, 200438, China; c Department of Rehabilitation Medicine, Chengdu Second People's Hospital, Chengdu, 610000, China; d Department of Rehabilitation Medicine, The First Affiliated Hospital of Xiamen University, School of Medicine, Xiamen University, Xiamen, 361003, China; e Department of Acupuncture and Moxibustion, Hubei University of Chinese Medicine, Wuhan, 430070, China; f Department of Spinal Surgery, The Second Affiliated Hospital of Guangzhou Medical University, Guangzhou, 510260, China

**Keywords:** lncRNA H19, miR-148a, Osteoarthritis, Mechanical load, Exercise, Autophagy, Pyroptosis

## Abstract

**Objective:**

Investigating whether mechanosensitive lncRNA H19 can directly target miR-148a to alleviate cartilage damage in post-traumatic osteoarthritis (PTOA).

**Methods:**

Thirty-two female rats were randomly divided into four groups: Sham-operated group (Sham group, n = 8), treadmill running group (R group, n = 8), anterior cruciate ligament transection (ACLT) group (ACLT group, n = 8), and ACLT + treadmill running group (ACLT + R group, n = 8). Histological evaluation was performed to observe the pathological changes in the cartilage of the rat knee. Micro-CT was performed to detect the bone morphological changes in the subchondral bone. RT-qPCR and Western-Blot were performed to detect changes in mRNA and protein levels of metabolic and inflammatory factors as well as changes in the expression of lncRNA H19 and miR-148a in cartilage. The Flexcell 5000™ Tension System was used to further validate that lncRNA H19 has mechanosensitivity *in vitro*. Finally, cell transfection techniques were used to knock down the expression of lncRNA H19 in chondrocytes to validate the regulatory role of lncRNA H19/miR-148a in cartilage metabolism.

**Results:**

ACLT combined with treadmill running aggravated the abnormal hyperplasia of subchondral bone in the lateral tibial plateau of the rat knee joint, disturbed the balance of cartilage metabolism, induced cartilage inflammatory response and chondrocyte pyroptosis, which eventually led to cartilage damage and PTOA. Importantly, we found that the expression of lncRNA H19 was significantly downregulated in the cartilage of the ACLT + R group. Bioinformatics analysis revealed that miR-148a may be a direct target of lncRNA H19. Subsequently, we focused on the mechanosensitive of lncRNA H19. Subsequently, moderate-intensity mechanical tension stress reversed the expression of lncRNA H19 and autophagy-related factors in inflammatory chondrocytes, while miR-148a showed an opposite expression trend, demonstrating that mechanosensitive lncRNA H19 may be involved in regulating the chondrocyte inflammatory response by targeting miR-148a and activating autophagy. Cell transfection experiments revealed that lncRNA H19 knockdown upregulated miR-148a expression and significantly inhibited the autophagy level of chondrocytes without significant alteration of chondrocyte pyroptosis, which in turn exacerbated the inflammatory response of chondrocytes.

**Conclusions:**

Mechanosensitive lncRNA H19 can promote chondrocyte autophagy rather than pyroptosis by targeting miR-148a, thus alleviating cartilage damage in PTOA. LncRNA H19 may be a potential therapeutic target for PTOA.

## 1 Introduction

Osteoarthritis (OA) is a chronic degenerative joint disease characterized by synovial inflammation, cartilage degeneration, vascular invasion, and subchondral bone remodeling. The initiation and progression of OA are influenced by multiple factors, including abnormal mechanical load, joint trauma, genetics, gender, age, and obesity [1]. Patients with OA typically experience joint pain, stiffness, dysfunction, and even joint deformity, resulting in disability. Epidemiological studies show that approximately 3.14 billion people worldwide suffer from OA, with the elderly accounting for more than 10 % [2], which places a serious financial burden on the patient's family and society. Currently, the pathogenesis of OA is not fully understood. The degeneration of articular cartilage is a key component leading to the formation of OA. The internal environment of the joint is gradually altered by various causes (metabolic, biochemical, biomechanical, etc.) [3]. Articular cartilage is composed of chondrocytes and extracellular matrix (mainly collagen fibers). As there are no nerves or blood vessels in cartilage, cartilage repair is extremely difficult once injury occurs. Articular cartilage is flexible and pliable, cushioning pressure loads and transferring them to the subchondral bone, adapting to various dynamic changes in the joint, maintaining joint motion and stability, and thus protecting the joint from injury [4]. Previous studies have shown that proper mechanical load facilitates normal growth and differentiation of chondrocytes, while excessive or insufficient mechanical load leads to imbalances in cartilage and subchondral bone metabolism [5]. Chondrocytes, the only cells in normal mature articular cartilage, are capable of producing and secreting collagen and proteoglycan to maintain normal articular cartilage homeostasis and metabolic balance [6]. The number and activity of chondrocytes in the articular cartilage of OA patients is significantly reduced. The ability of low-active chondrocytes to synthesize and secrete collagen is further reduced, resulting in poorer quality, less elastic, and less resistant articular cartilage and ultimately impaired articular cartilage and subchondral bone function [[7], [8], [9]]. Studies have shown that increasing the activity and number of chondrocytes can partially mitigate the progression of OA. Therefore, investigating the molecular mechanisms of OA chondrocyte metabolism to improve chondrocyte activity is important to explore the initiation and progression of OA and to develop novel therapeutic strategies to treat it.

Autophagy is a self-degradative process that is important for balancing sources of energy at critical times during development and in response to cell stress. During autophagy, parts of the cytoplasm and intracellular organelles are sequestered within characteristic autophagic vacuoles and are subsequently degraded by lysosomes [10]. In most circumstances, autophagy promotes cell survival by adapting cells to stress conditions, but at the same time, this process has also been considered as a non-apoptotic cell death program [11]. Recent studies have shown that autophagy occurs in the course of many diseases, such as cancer [12], Alzheimer's disease [13], and OA [14]. Alterations in autophagy levels can lead to abnormal homeostasis of osteocyte, chondrocyte and other cells, leading to a variety of diseases. Almonte-Becerril et al. [11] demonstrated that autophagy probably could be activated as an adaptive response that avoids cell death in early stages of OA. Whereas, this process also could be conjunctly activated with apoptosis as an alternative pathway to cellular demise in late stages of OA. In addition, studies have shown that some autophagy-inducing agents have a protective effect on OA. Among them, Tougu Xiaotong capsule inhibits tidemark replication and cartilage degradation by regulating chondrocyte autophagy and it could be a potential therapeutic agent for the reduction of cartilage degradation that occurs in OA [15]. Recent studies showed that rapamycin activates autophagy in human chondrocytes preventing the development of OA *in vitro*, while the systemic and/or intra-articular injection of rapamycin reduces the severity of experimental OA in vivo [16], representing a potential therapeutic approach to prevent OA. However, the specific regulatory mechanism of autophagy in the development of OA remains unclear. Therefore, it is of great significance to further study and explore the targeted genes and pathways related to autophagy regulation in the process of OA.

Long non-coding RNAs (lncRNAs) are a class of RNA molecules more than 200 nucleotides in length, which are widely involved in regulating cell development, proliferation, and apoptosis [17,18]. Previous studies have suggested that lncRNAs are capable of acting as signaling, scaffolding, guidance and decoy molecules involved in the regulation of coding genes [19]. The specific regulatory mechanisms include: regulating transcription by binding DNA or RNA through base complementary pairing and interfering with their target genes [20]; binding to proteins to influence the formation of protein polymers to play a regulatory role [21]; regulating the expression of downstream genes through sponge adsorption of microRNAs (miRNAs) [22]; and recruiting chromatin modification factors to change the level of chromatin modification, thus participating in gene regulation [23]. In fact, these several regulatory mechanisms of lncRNAs do not exist independently, but are interrelated and influenced by each other, which are closely related to a variety of human diseases. Accumulating evidence suggests that the pathogenesis of OA is very closely related to lncRNAs [24]. Xing et al. [25] used microarray analysis to compare the expression of lncRNAs in OA cartilage with that in normal cartilage and found that 73 lncRNAs were upregulated and 48 lncRNAs were downregulated in OA cartilage, with 21 lncRNAs, including lncRNA H19, upregulated more than 2-fold above normal levels. It has been demonstrated that lncRNAs may be extensively involved in the regulation of pathological changes in OA, including cell proliferation and apoptosis [26,27], inflammatory response [24], and angiogenesis [28]. The relationship between lncRNA H19 and OA lesions was first verified by Steck et al. [29]. This study showed that lncRNA H19 can affect cartilage metabolism by targeting miR-675, suggesting that lncRNA H19 may be a potential target for stimulating cartilage repair. Subsequently, multiple studies demonstrated that lncRNA H19 may be involved in regulating the metabolic processes of chondrocytes *in vitro* [[30], [31], [32]]. It is widely believed that lncRNA H19 may play a regulatory role in OA by indirectly regulating chondrocyte metabolism and cartilage matrix degradation, mainly through targeting miRNAs [[33], [34], [35], [36]]. Therefore, exploring the target genes of lncRNA H19 and verifying its expression changes in OA are crucial to deeply investigate the effects of lncRNA H19 on OA lesions and to explore potential therapeutic targets for OA.

Mechanical load refers to the forces to which bones and joints are subjected during physiological activities such as movement, weight-bearing, and postural control. Moderate mechanical load stimulates chondrocyte synthesis of type II collagen (COL-II) and reduces matrix metalloproteinase (MMP)-13 production, thereby regulating processes such as chondrocyte senescence and apoptosis to protect against cartilage damage [37,38], while too high or too low load stimulation accelerates chondrocyte senescence, increases apoptosis and promotes the progression of OA [39]. Previous studies have shown that under prolonged exposure to repetitive or excessive mechanical stress, micro-injuries to bones and joints can develop and gradually accumulate. This long-term microdamage induces an inflammatory response in chondrocytes, accelerating chondrocyte apoptosis and degeneration of articular cartilage, ultimately leading to OA [[40], [41], [42]]. Li et al. [43] established a rat model of disuse osteoporosis (DOP) by tail suspension and found that mechanical stress unloading decreased lncRNA H19 expression and promoted Dkk4 expression, which inhibited the Wnt signaling and ultimately led to reduced bone formation and DOP, suggesting that lncRNA H19 may be regulated by mechanical stress. Similarly, in our previous study, an exercise-induced traumatic OA mouse model was constructed by high-intensity treadmill running. We found that high-intensity treadmill running downregulated lncRNA H19 expression in cartilage. Subsequently, moderate-intensity treadmill running alleviated cartilage damage in OA mice and promoted lncRNA H19 expression, which further revealed that lncRNA H19 is a mechanosensitive lncRNA and plays an essential regulatory role in the initiation and development of exercise-induced traumatic OA [44]. However, our preliminary study found that high-intensity treadmill running only induced cartilage damage and did not show a significant OA phenotype. There is a need to further design animal models to confirm the regulatory mechanism of mechanosensitive lncRNA H19 in OA lesions, which may provide a basis for appropriate exercise to alleviate OA as well as a potential target for OA treatment. Accumulating evidence suggests that lncRNAs are able to directly target miRNAs to play important regulatory roles in a variety of bone metabolic diseases, including osteoporosis (OP) and OA [45]. In this study, we first revealed that lncRNA H19 can promote chondrocyte autophagy rather than pyroptosis by targeting miR148a in PTOA. Moderate-intensity mechanical stretching partially reversed the decrease in chondrocyte autophagy caused by knockdown of lncRNA H19, thereby alleviating chondrocyte injury, which also provides new insights into the mechanisms by which exercise alleviates PTOA.

## 2 Materials and methods

### 2.1 Animal experiment

This study was carried out in strict accordance with the recommendations from the Ethical Committee of Beijing Sport University on the Care and Use of Animal Subjects in Research (Approval Number: 2023026A), following the protocols approved by the committee. Thirty-two 12-week-old female Sprague-Dawley (SD) rats were purchased from Beijing Huafukang Biotechnology Co. All rats were housed in an SPF-grade animal laboratory environment using standard rat cages and allowed to move freely within the cage. The rats were fed a national standard solid diet and water at a controlled temperature and relative humidity of (22 ± 2)°C and 55%–75 % with a normal circadian rhythm. All rats were acclimatized for 1 week before formally starting to receive the intervention. Rats were weighed every Monday. The rats were randomly divided into four groups: Sham-operated group (Sham group, n = 8), treadmill running group (R group, n = 8), anterior cruciate ligament transection (ACLT) group (ACLT group, n = 8), and ACLT + treadmill running group (ACLT + R group, n = 8).

### 2.2 Surgical protocol

The rats were fasted for 24 h prior to the surgery and provided with water only. All surgical instruments were sterilized in an autoclave according to a predetermined process. Surgical rats were anesthetized by inhalation of isoflurane and placed in the supine position on the operating table. No reflexes after pinching the rat's toes indicated adequate anesthesia to perform the procedure. Based on the white medial collateral ligament for surgical positioning, the skin of the lateral knee joint of the rat was incised with a surgical blade, and the joint capsule was subsequently opened to reveal the femoral condyle, tibial plateau, and ACL. The knee is maintained in flexion to fully expose the ACL. Rats in the ACLT group and ACLT + R group were sutured to the joint capsule and skin after ACL dissociation. Rats in the Sham and R groups were sutured to the joint capsule and skin after ACL exposure without dissociating the ACL. The rats were given intramuscular penicillin immediately after surgery to prevent infection.

### 2.3 Treadmill running program

Rats in the ACLT + R group began treadmill acclimatization training 1 week after surgery. Rats in the R group also received treadmill running training at the same time as rats in the ACLT + R group. The treadmill running training program was developed based on the previous research experience of our group [44,46,47]. As shown in Table 1, treadmill acclimatization training lasted 1 week, with 30 min of running at a speed of 10 m/min from the first to the third day. Rats were run at a speed of 12 m/min for 40min on the fourth and fifth days. Rats were run at a speed of 14 m/min for 50 min on the sixth and seventh day. Subsequently, an 8-week formal treadmill training program was performed. For the first week of formal treadmill running, the rats ran at a speed of 16 m/min for 60min. Subsequently, the speed of the treadmill was increased by 2 m/min per week until it reached 24 m/min. The slope of the treadmill is 30°. During the formal treadmill running training, the speed of the treadmill gradually increased from 10 m/min to the target speed in the first 10min, maintaining the target speed for 45min in the middle, and the speed of the treadmill gradually decreased from the target speed to 0 m/min in the last 5min. Rats in the Sham and ACLT groups were housed in cages and allowed to move freely. After eight weeks of formal treadmill running training, all groups of rats were sacrificed. Anesthetize the rats by isoflurane inhalation. Open the rat's thorax to obtain blood at the apical part of the heart.

**Table 1:** Treadmill running program.

Stages	Time	Speed	Duration	Slope
1 week of acclimatization training	day 1–3	10 m/min	30 min/d	0°
	day 4–5	12 m/min	40 min/d	0°
	day 6–7	14 m/min	50 min/d	0°
8 weeks of formal treadmill running training	week 1	16 m/min	60 min/d	30°
	week 2	18 m/min	60 min/d	30°
	week 3	20 m/min	60 min/d	30°
	week 4	22 m/min	60 min/d	30°
	week 5–8	24 m/min	60 min/d	30°

### 2.4 Micro-CT scan

The intact knee joints of rats were placed in paraformaldehyde fixative for 48 h and then transferred to 70 % alcohol solution and stored in a refrigerator at 4 °C. The Skyscan 1276 scanning device was used to scan the rat knee joint. The scanning parameters were as follows: Camera Pixel Size (um) = 9.01; Source Voltage (kV) = 69; Source Current (uA) = 100; Image Pixel Size (um) = 9.92. After scanning, the highest point of the tibial plateau was selected as the starting position downward for 200 layers. The medial and lateral sides of the tibial plateau were used as the regions of interest for reconstruction, respectively. The analysis of bone microstructural parameters, including BV/TV, Tb.Th, Tb.N and Tb. Sp, was performed using CTan software.

### 2.5 Hematoxylin-eosin staining and saffron O solid green staining

After micro-CT scanning, the knee samples of each group of rats were transferred to EDTA decalcification solution for decalcification. The decalcification solution was changed every 2–3 days. Completion of decalcification is indicated when the bone tissue can be easily pierced using the syringe tip. The samples were then dehydrated, transparent, waxed, embedded in a tissue wax block, and finally sectioned (section thickness of 4 μm). Hematoxylin-eosin staining and Saffron O solid green staining were routinely performed. After staining, the sections were observed and photographed under microscopic magnifications of 20× and 200X, respectively. The stained sections of rat knee joints were scored by two independent experienced researchers according to the criteria of Mankin's score and OARSI score. The mean of the scores was taken as the final score.

### 2.6 Immunohistochemical and immunofluorescent staining

Immunohistochemical staining was performed according to routine procedures. Briefly, paraffin sections were routinely dewaxed. Antigen was repaired overnight at 60 °C using sodium citrate buffer (Beyotime, P0083). Rinse 3 times using PBS. Triton 100 was added dropwise in 10 min and rinsed 3 times using PBS. Subsequently, the closure solution (Beyotime, P0260) was used for 1 h and rinsed 3 times with PBS. Primary antibodies (anti-COL-II (Proteintech, 28459-1-AP) and *anti*-MMP-13 (absin, abs120041)) were added dropwise to the sections and incubated overnight at 4 °C in the refrigerator. The secondary antibody was added dropwise after PBS × 3 washes and incubated for 1–2 h at room temperature. The steps for immunofluorescence staining are similar to those for immunohistochemical staining. After the primary antibody incubation (*anti*-Beclin1 (abmart, T55092) and *anti*-LC3B (abmart, T55992)), the incubation was carried out using a fluorescent secondary antibody for 1–2 h at room temperature protected from light. Finally, the slices were sealed using a DAPI-containing sealer. Moreover, immunohistochemical staining was performed according to the operating instructions of the Immunohistochemistry Kit and DAB Color Development Kit. Hematoxylin stain was used to stain the nuclei of the cells. Analysis was performed using ImageJ to assess the positive expression of proteins between the groups.

### 2.7 RT-qPCR

Total cartilage tissue RNA was extracted using Trizol reagent. Briefly, the cartilage attached to the femoral condyle and tibial plateau is peeled off after removing the localized ligaments and other excess connective tissues from the knee joint. Cartilage tissue was placed in a mortar. The cartilage tissue was ground and pulverized using liquid nitrogen and transferred to 1.5 ml EP. 1 ml of Trizol was added to a 1.5 ml EP tube and followed according to the instructions to extract total RNA. The takara PrimeScript RT reagent Kit (Takara, RR037A) was used to reverse transcribe RNA into cDNA according to the instructions. The takara SYBRGreen kit (Takara, RR820A) was used for real-time fluorescent quantitative amplification of target genes. U6 was used as an internal reference for miRNA and β-actin was used as an internal reference for other genes. The 2^−ΔΔCT^ method was used to analyze the data. All primers involved were designed using Primer premier 5.0 software (As shown in Table 2).

**Table 2:** Primer sequences.

Genes	Primer sequence (5′-3′)
lncRNA H19	Forward: 5′- GCTCCACTGACCTTCTAAAC -3′ Reverse: 5′- ACGATGTCTCCTTTGCTAAC -3′
miR-148a	Forward: 5′- TCAGTGCACTACAGAACTTTG -3′
β-actin	Forward: 5′-CTGTCCCTGTATGCCTCTG-3′ Reverse: 5′-ATGTCACGCACGATTTCC-3′
MMP-9	Forward: 5′-TTGACAGCGACAAGAAGTGG-3′ Reverse: 5′-GCCATTCACGTCGTCCTTAT-3′
MMP-13	Forward: 5′-ACGTGTGGAGGTGAGGCATCC-3′ Reverse: 5′-GCAGAAGGCAGACCGCAATGG-3′
COL-II	Forward: 5′-CATGACCTCGTGATGAACGTG T-3′ Reverse: 5′-CGGGTGAGGACGTTTACAAAG-3′
NLRP3	Forward: 5′- GTGCAGATCCTAGGTTTCTCTG-3′ Reverse: 5′- CAGGATCTCATTCTCTTGGATC-3′
Caspase-1	Forward: 5′-ACGCCTTGCCCTCATAAT-3′ Reverse: 5′-TCTAATACATCTGGGACTTCTT-3′
IL-1β	Forward: 5′-CAGCTTTCGACAGTGAGGAGA-3′ Reverse: 5′-TGTCGAGATGCTGCTGTGAG-3′
IL-18	Forward: 5′-GACCTGGAATCAGCAACTTTGG-3′ Reverse: 5′-GCCTCGGGTATTCTGTTATGGA-3′
TNF-α	Forward: 5′- AGGCTGCCCCGACTACGT -3′ Reverse: 5′- GACTTTCTCCTGGTATGAGATAGCAAA -3′
U6	Forward: 5′- AACGCTTCACGAATTTGCGT -3′ Reverse: 5′- GACTTTCTCCTGGTATGAGATAGCAAA -3′

### 2.8 Western-Blot

As with the RNA extraction step, the cartilage tissue samples are ground and pulverized using liquid nitrogen. Subsequently, RIPA lysate and PMSF protease inhibitor were added, and total proteins were extracted by centrifugation at 12000 rpm for 20 min. After measuring the protein concentration with the BCA kit to homogenize the protein concentration, the protein loading buffer was added and cooked at 100 °C for 5 min. The proteins were separated using SDS-PAGE gel electrophoresis and then transferred to PVDF membranes. Subsequently, closure was performed using 3–5% BSA solution. The corresponding primary antibody was added at 4 °C and incubated overnight. Wash three times with TBST and add the corresponding secondary antibody and incubate at room temperature for 1–2 h. Finally, the chemiluminescent solution was used to develop the image. Anti-COL-II (Proteintech, 28459-1-AP), *anti*-ACAN (Proteintech, 13880-1-AP), *anti*-MMP-13 (absin, abs120041), *anti*-β-actin (abmart, TP70573), *Anti*-IL-18 (abmart, TD6252), *anti*-NLRP3 (abmart, T55651), *anti*-Caspase-1 (abmart, TU410369), and *anti*-β-tubulin (abclonal, A12289) were used as primary antibody. The grayscale values were counted using Image J software.

### 2.9 Primary chondrocyte extraction

Newborn rats within seven days of birth were dislocated and executed by cervical dislocation. Newborn rats were immersed in a 75 % alcohol solution for 10 min. The knee cartilage was removed using surgical instruments after autoclaving. The cartilage tissue was rinsed three times with the PBS solution containing 1 % penicillin and streptomycin. 0.25 % trypsin was added for digestion in a cell incubator at 37 °C for 30 min. The supernatant was discarded and washed 2–3 times with complete medium. 0.1 % type II collagenase solution was used to continue digestion of cartilage tissue for 12–16 h. Subsequently, the digested suspension was centrifuged. The supernatant was discarded and 5 ml of DMEM medium containing 10 % fetal bovine serum (FBS) was added and incubated in a cell culture incubator at 37 °C with 5 % CO_2_. The complete culture solution was replaced every other day. When chondrocytes reached 80 %–90 % fusion, passaging was performed. p1-p2 chondrocytes were used for subsequent formal experiments.

### 2.10 Toluidine blue staining

After the chondrocytes reached 80 % fusion, the complete culture medium was discarded. PBS was used to wash 3 times. Subsequently, chondrocyte morphology was fixed using paraformaldehyde for 15–20 min. PBS was used to wash 3 times. Toluidine blue solution was added for 10–20min. Through real-time observation of dyeing degree, toluidine blue solution was abandoned in time to stop dyeing. PBS was used to wash off excess toluidine blue solution. After drying, check the staining under an optical microscope and collect images.

### 2.11 CCK-8

The primary chondrocytes of P2 generation rats were prepared into a single cell suspension and added to a 96 well plate with 5000 cells per well. The experimental groups were divided into five groups: 0 ng/ml, 0.1 ng/ml, 1 ng/ml, 10 ng/ml, and 20 ng/ml. Each group is equipped with 5 parallel secondary holes. The 96 well plate was placed in the cell incubator for 24 h 10 % CKK-8 solution was added to each well and incubated for 1 h. Finally, Microplate reader was used to measure the optical density (OD) values of each well at 450 nm.

### 2.12 Bioinformatics analysis

The sequence and annotation information of lncRNA-H19 was queried through the NCBI database to understand the biological function, expression pattern, and regulatory mechanism of this gene. The sequence of lncRNA-H19 was predicted to clarify the structure of its miRNAs containing target sites. The target gene prediction of lncRNA H19 was performed using target site analysis software such as Diana tools database, ENCORI database, miRcode database, and miRDB database, respectively. The target genes that could be predicted by all four databases were screened as candidate target genes. The intersection of the screening results from the four databases was used as the set of genes for further study. The predicted results were plotted on the venny diagram.

### 2.13 Mechanical load experiment *in vitr*o

Rat primary chondrocytes were inoculated into the mechanically stretched custom-made six-well plates. The chondrocytes were subjected to tension stress using the Flexcell-5000™ Tension System. Stretching forces of 10 % deformation strength were used with 1 h, 2 h, and 4 h intervention times.

### 2.14 Cell transfection experiment *in vitr*o

Knockdown of lncRNA H19 expression in primary chondrocytes using electrotransfer technology. Prepare 1 × 10^6^ P1 generation primary chondrocytes. Wash twice with PBS. Add 100 μl of electrotransfection buffer from Lonza P3 primary cell transfection kit and add 2 μg of plasmid. A pipette gun was used to pipette well. Turn on the LONZA 4D-Nucleofector ahead of time and select the corresponding chondrocyte electrotransformation program. Transfer the cell mixture to the electrotransformation cup to start electrotransformation. At the end of the electrotransformation, 500 μl of cell complete culture solution was added to the electrotransformation cup. The cell mixture was then pipetted out and transferred to a 6-well plate. After 8 h, replace the cell culture medium. The chondrocytes were divided into Vector negative control group (Vector NC group), Vector+10 μg/ml LPS group (Vector LPS group), knockdown lncRNA H19 group (Si-H19 group), and knockdown lncRNA H19 + 10 μg/ml LPS group (Si-H19 LPS group). After transfection, total RNA was extracted and the expression of lncRNA H19 was measured to find the most appropriate transfection concentration for subsequent experiments.

### 2.15 Transmission electron microscopy

The cell samples for transmission electron microscopy were handed over to Sevier (Wuhan, China) Biotechnology Co. for sample preparation and scanning. Briefly, chondrocytes were fixed using glutaraldehyde after centrifugation collection. Then pre-embedding was performed using Agarose. Subsequently, after post-fix, dehydrate, resin penetration and embedding, polymerization, ultrathin section, and staining, the samples were observed and photographed under transmission electron microscope.

### 2.16 Dual-luciferase reporter assay

The psiCHECK-2 carrier profile (Fig. S3) was used in this experiment. The T7 promoter is followed by a multiple cloning site (MCS) region. Two enzymatic sites, XhoI and NotI, were used to insert the target fragment. Primer sequences were synthesised by Shenggong Biotechnology (Shanghai) Co., Ltd. PCR amplification was performed after the restriction. Subsequently, DH5a receptor cells were used for transformation culture. Single bacteria were picked for bacteriological sequencing after overnight incubation. Then, cell transfection experiments were performed using 293T cells. Briefly, 10 μl of DMEM was mixed thoroughly with 0.1 μg of WT/MUT destination plasmid and 5 pmol of microRNA/Negative.Control (N.C) at room temperature (Solution A). Meanwhile, 10 μl of DMEM was mixed thoroughly with 0.2 μl of lipo 3000 transfection reagent (Solution B). Solution A and B were mixed and left to stand at room temperature for 15 min, and then added to the cell culture plate. The complete medium was replaced after 6 h. Luciferase assay was performed 48 h after transfection. The Promega Dual-Luciferase system was used on multifunctional enzyme markers with chemiluminescent modules to detect the luminescent signal of fluorophore enzymes and record the Renilla luciferase value, which is the reporter gene luminescence value.

### 2.17 Data analysis

The data from each group were analyzed using SPSS 20.0 software. GraphPad Prism 8 was used to graph statistical data. Results are expressed as mean ± standard deviation ($\bar{X}$ ±*S*). The groups were compared using one-way ANOVA (One way ANOVA) and the LSD method was used to compare the groups with each other. P < 0.05 indicates a significant difference.

## 3 Results

### 3.1 Pathological changes of articular cartilage in rats

**Fig. 1:**
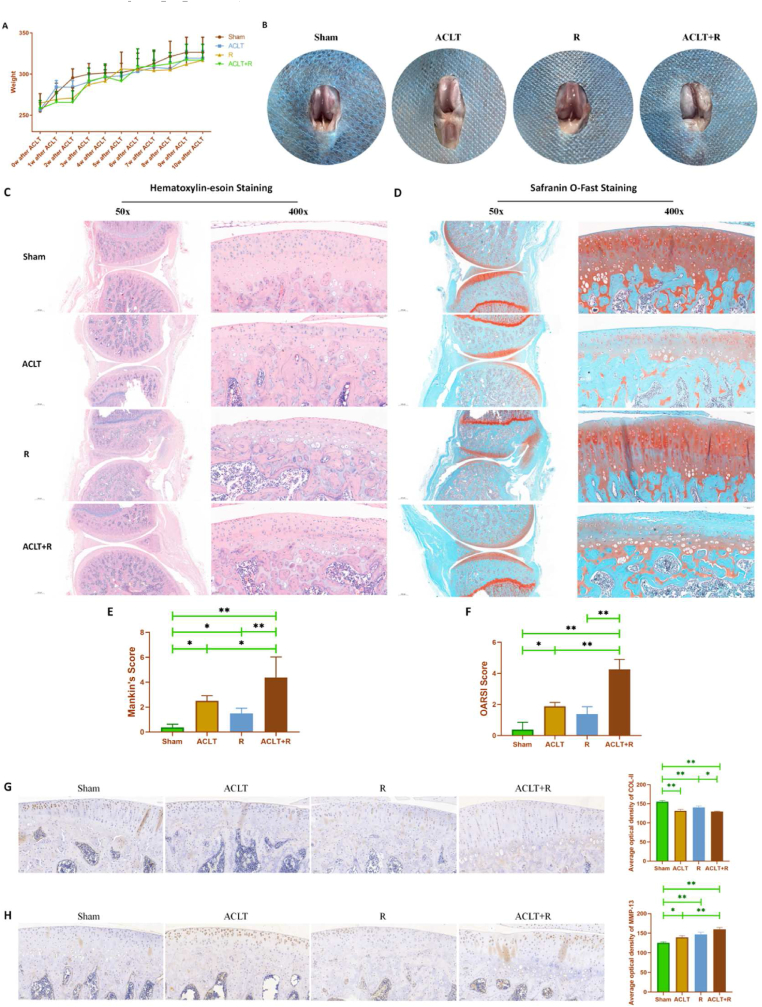
**Pathological changes of articular cartilage in rats.** (A: changes in body weight of rats in each group. B: gross observation of knee cartilage in each group of rats. C: Hematoxylin-eosin staining of knee sections of rats in each group. D: Safranin O and Fast Green staining of knee sections of rats in each group. E: Mankin's score of knee sections of rats in each group. F: OARSI scores of knee sections of rats in each group. G: COL-II protein expression in knee sections from various groups of rats by immunohistochemistry. H: MMP-13 protein expression in knee sections from various groups of rats by immunohistochemistry. Sham group: Sham-operated group; ACLT group: anterior cruciate ligament transection surgery group; R group: treadmill running group; ACLT + R group: anterior cruciate ligament transection surgery plus treadmill running group, ∗P < 0.05, ∗∗P < 0.01).

Rats were weighed once a week. As shown in Fig. 1A, the body weight of rats in the Sham group showed an increasing trend with age. Although there was a tendency for the body weight of the operated rats to decrease within 3w postoperatively (no significant difference), the body weight of the rats in all groups was basically the same after 4 w postoperatively. In addition, the trend of body weight change of the rats in the R group was also generally consistent with that of the operated rats. The rats in each group were sacrificed at the end of the treadmill running intervention. The knee capsule of the rats was opened for macrophotography. As shown in Fig. 1B, the articular surfaces of the Sham group were sharply colored and structurally intact. The articular cartilage surfaces of the rats in the ACLT and R groups were slightly whitish in color, although there was no obvious visible wear. The articular cartilage surfaces of the rats in the ACLT and R groups were slightly whitish in color, although there was no obvious visible wear, whereas the articular cartilage in the ACLT + R group was whitish in color, significantly swollen, with a bumpy surface and obvious wear. Moreover, the ACL was clearly visible in the Sham and R group rats, but not in the ACLT and ACLT + R group rats.

Paraffin sections of the knee joint of rats in each group were stained with hematoxylin-eosin and saffron O solid green. As shown in Fig. 1C, the chondrocytes in the Sham group were basically uniform in size and more regularly distributed. The overall cartilage surface was smooth; the chondrocytes in the ACLT group were locally aggregated, and the non-aggregated areas were scattered. The cartilage surface showed mild wear and unevenness; the chondrocyte distribution in the R group was basically neat, with a little discontinuity. The degree of cartilage wear was lighter compared with the ACLT group; the distribution of chondrocytes was uneven in the ACLT + R group. There were multiple fissures in the cartilage layer with serious loss, suggesting that there was severe cartilage wear. The results of the Safranin O and Fast Green staining were in general agreement with the HE staining results (Fig. 1D). In the Sham group, the cartilage was overall flat and smooth, with uniform distribution and no abnormal morphology or interruption; in the ACLT group, the cartilage showed localized fibrosis and low cell nuclei density in individual areas, suggesting cartilage degeneration; in the R group, the distribution of cartilage was basically normal, but compared with the Sham group, the density of cell distribution was reduced, suggesting slight degeneration of cartilage; in the ACLT + R group, the distribution of cartilage was more discontinuous, with multiple fibrosis-like changes, significantly increased invasion of solid green-stained tissues, and fissures, suggesting more serious degeneration of cartilage. Moreover, as shown in Fig. 1E and F, the Mankin's score (p < 0.05) and OARSI score (p < 0.05) of the knee joint of the rats in the ACLT group were both significantly increased compared to the Sham group, suggesting that the rat model of PTOA was successful. Furthermore, the Mankin's score (p < 0.05) and OARSI score (p < 0.001) in the ACLT + R group were significantly higher than those in the ACLT group. Further, immunohistochemical staining was performed on the knee sections of each group of rats. Compared with the Sham group, the protein expression of the chondrocyte anabolic marker COL-II was decreased in the ACLT, R, and ACLT + R groups (Fig. 1G), whereas the protein expression of the chondrocyte catabolic marker MMP-13 was significantly upregulated (Fig. 1H). In particular, the protein expression of MMP-13 in the ACLT + R group was significantly higher than that in the ACLT group (p < 0.01).

### 3.2 Bone morphological changes of subchondral bone in rats

**Fig. 2:**
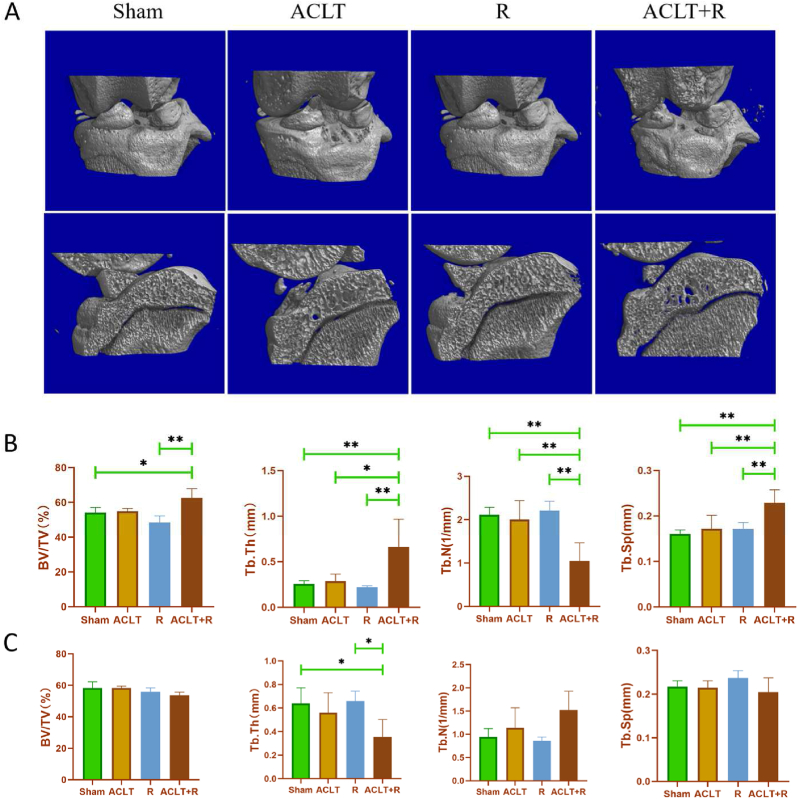
**Bone morphological changes of subchondral bone in rats.** (A: 3D reconstructions of the lateral and medial tibial plateaus in each group of rats. B: Changes in bone microstructural parameters of the lateral tibial plateau in each group of rats. C: Changes in bone microstructural parameters of the medial tibial plateau in each group of rats. Sham group: Sham-operated group; ACLT group: anterior cruciate ligament transection surgery group; R group: treadmill running group; ACLT + R group: anterior cruciate ligament transection surgery plus treadmill running group, ∗P < 0.05, ∗∗P < 0.01).

Micro-CT was used to evaluate the morphological changes in the subchondral bone of the tibia. Bone microstructure reconstruction and analysis were performed on the medial and lateral tibial plateau, respectively (Fig. 2A). Fig. 2B demonstrates the changes in bone microstructural parameters in the lateral tibial plateau of rats in each group. BV/TV in the ACLT + R group was significantly higher than that in the Sham group (p < 0.05) and the R group (p < 0.01). Tb.Th and Tb. Sp in the ACLT + R group were significantly higher than those in the Sham, ACLT, and R groups, while Tb.N in the ACLT + R group was significantly lower than those in the Sham, ACLT, and R groups (p < 0.01). Interestingly, the bone morphology of the medial tibial plateau is not altered as much as that of the lateral tibial plateau (Fig. 2C). The medial tibial plateau showed significant differences in only one index, Tb.Th, in the comparison between groups. Tb.Th was significantly lower in the ACLT + R group than in the Sham and R groups (p < 0.05).

### 3.3 Key genes and protein expression changes of articular cartilage in rats

**Fig. 3:**
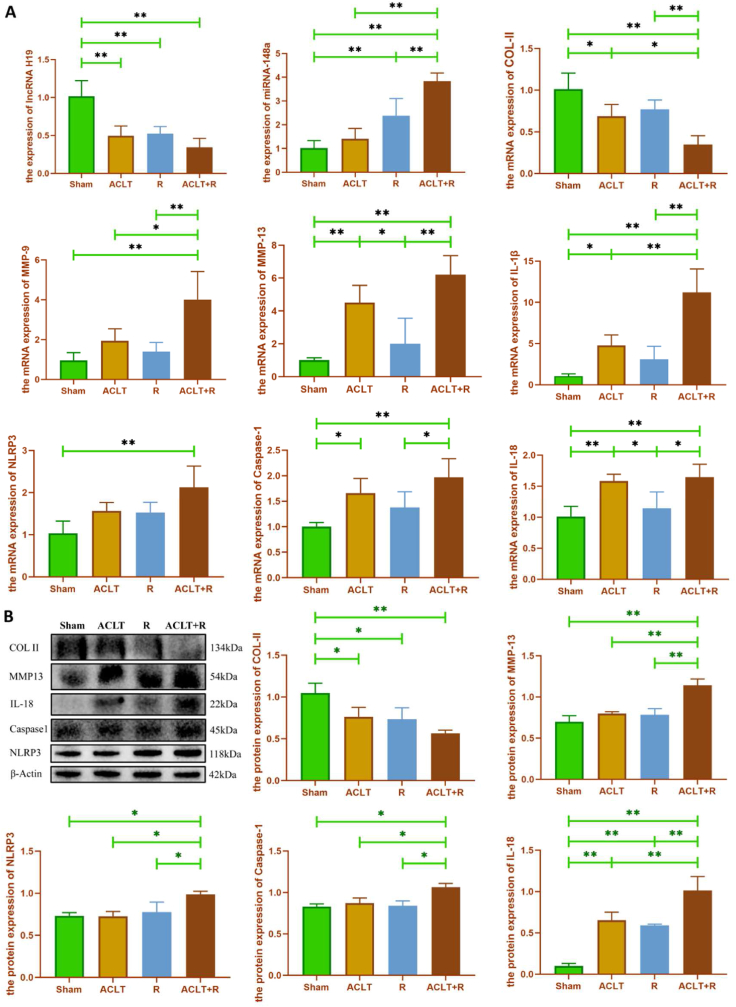
**Key genes and protein expression changes of articular cartilage in rats.** (A: Changes in lncRNA H19, miR-148a, and metabolic and inflammation-related marker gene expression in cartilage. B: Changes in protein expression of metabolic and inflammation-related markers in cartilage. Sham group: Sham-operated group; ACLT group: anterior cruciate ligament transection surgery group; R group: treadmill running group; ACLT + R group: anterior cruciate ligament transection surgery plus treadmill running group, ∗P < 0.05, ∗∗P < 0.01).

Total RNA and total protein from rat articular cartilage were extracted to detect changes in gene and protein expression of cartilage anabolic, catabolic, and inflammatory markers. As shown in Fig. 3A, compared with the Sham group, the expression of lncRNA H19 in cartilage was significantly lower in the ACLT, R, and ACLT + R groups (p < 0.01), while the expression of miR-148a was significantly higher (p < 0.01). Importantly, the expression of miR-148a was significantly higher in the ACLT + R group than in the ACLT and R groups (p < 0.01), while the expression of lncRNA H19 had a decreasing trend compared to the ACLT and R groups (p > 0.05). In addition, the expression of the anabolic marker COL-II mRNA was significantly decreased in the ACLT + R and ACLT groups compared to the Sham group, while the expression of the catabolic markers MMPs and IL-1β mRNA, a marker of inflammation, was significantly increased. The changes in protein expression of COL-II and MMP-13 were generally consistent with the changes in their mRNA expression (Fig. 3B). Moreover, the expression of key genes and protein markers related to pyroptosis was also detected. We found that only the expression of NLRP3 in the ACLT + R group was significantly higher than that in the Sham group (p < 0.01), while NLRP3 expression in the ACLT or R groups was not significantly increased compared with that in the Sham group (p > 0.05). The expression of IL-18 in both the ACLT + R and ACLT groups was significantly higher than that in the Sham group (p < 0.01), while the expression of IL-18 in the R group was not significantly different from that in the Sham group (p > 0.05). Further, protein expression of autophagy markers in rat articular cartilage was detected by immunofluorescence (Fig. S2). We found that compared to the Sham group, the protein expression of both Beclin1 and LC3B was significantly reduced in the ACLT and R groups, especially in the ACLT + R group, suggesting that the level of autophagy was reduced in the damaged articular cartilage.

### 3.4 Knockdown of lncRNA H19 inhibits autophagy rather than pyroptosis in chondrocytes

**Fig. 4:**
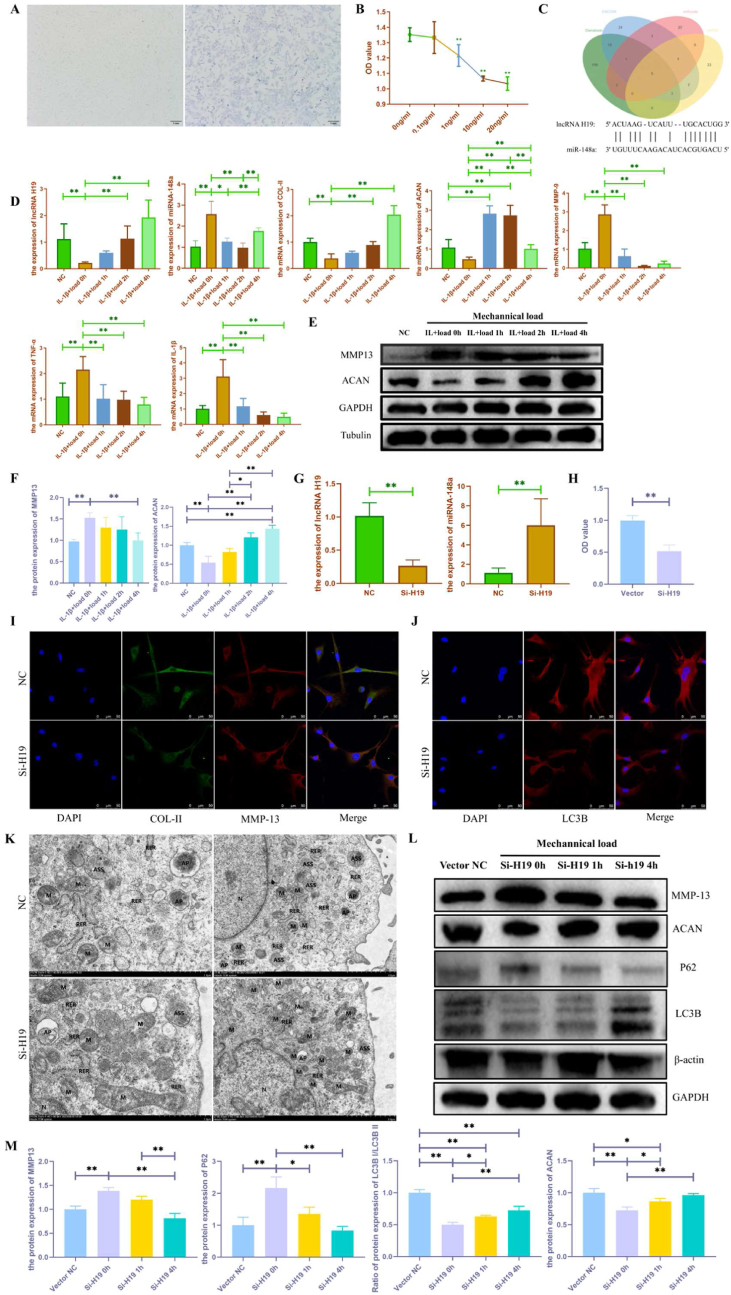
**Effect of moderate-intensity mechanical load on chondrocyte injury.** (**A**: Toluidine blue staining was used to identify chondrocytes. **B**: CCK-8 was used to evaluate the effect of IL-1β on chondrocyte activity. **C:** Bioinformatics analysis was used to predict the target genes of lncRNA H19. **D**: RT-qPCR was used to examine the effect of moderate-intensity mechanical load on the lncRNA H19, miR-148a, and the mRNA expression of chondrocyte metabolism and inflammatory factors in IL-1β-induced chondrocytes. **E-F**: Western Blot was used to examine the effect of moderate-intensity mechanical load on protein expression of MMP-13 and ACAN in IL-1β-induced chondrocytes. **G**: siRNA technology was used to knock down lncRNA H19 expression and detect miR-148a expression by q-PCR in chondrocytes. **H**: CCK-8 was used to detect chondrocyte activity after lncRNA H19 knockdown. **I-J**: Immunofluorescence was used to detect changes in chondrocyte metabolism and autophagy levels after lncRNA H19 knockdown. **K**: Transmission electron microscopy was used to detect changes in autophagy levels in chondrocytes after lncRNA H19 knockdown. Nucleus (N), Mitochondria (M), Rough endoplasmic reticulum (RER), Autophagosome (AP), Multivesicular body (Mvb), Autophagosome-lysosome (ASS), Golgi apparatus (Go). **L-M**: Western Blot was used to examine the effects of moderate-intensity mechanical stretch on autophagy and metabolism in chondrocytes with knockdown of lncRNA H19. NC group: negative control group; IL-1β+load 0 h group: 10 ng/ml IL-1β supplementation group; IL-1β+load 1 h group: IL-1β supplementation + 1 h moderate-intensity mechanical load group; IL-1β+load 2 h group: IL-1β supplementation + 2 h moderate-intensity mechanical load group, IL-1β+load 4 h group: IL-1β supplementation + 4 h moderate-intensity mechanical load group, ∗P < 0.05, ∗∗P < 0.01).

Primary chondrocytes were extracted from newborn rats. Toluidine blue staining was used to identify chondrocytes. The cations of toluidine blue can be stained by combining with acid, where two hair chromophores and two helper chromophores can stain the nucleus blue (Fig. 4A). The cell clusters are pavement-like and individually polygonal in light microscopy, which is typical of chondrocyte morphology. Then, the CCK-8 kit was used to assess the proliferative activity of chondrocytes under the intervention of the inflammatory factor IL-1β to determine the optimal concentration of IL-1β to use for follow-up experiments. As shown in Fig. 4B, a significant decrease in chondrocyte activity occurred at an IL-1β concentration of 1 ng/ml (P < 0.01). Combining the experience of our groups, we finally decided to use a concentration of 10 ng/ml, which may be a more stable induction concentration.

Subsequently, we proposed the hypothesis that lncRNA H19 can regulate chondrocyte injury by targeting miRNAs. Therefore, bioinformatics analysis was used to predict potential targets of lncRNA H19. Four databases, miRcode, miRDB, ENCORI, and Dianatools, were used for lncRNA H19 target gene prediction (Fig. 4C). The prediction results of the four databases were intersected. Finally, five miRNAs were identified as potential targets of lncRNA H19 (miR-301b, miR-148a, miR-130a, miR-130b, and miR-107). Furthermore, moderate intensity mechanical tension stress was applied to IL-1β-induced primary chondrocytes. The results of RT-qPCR showed that only miR-148a expression in chondrocytes was upregulated after IL-1β supplementation, while moderate-intensity mechanical load partially reversed the IL-1β-induced upregulation of miR-148a expression (P < 0.01), which was in contrast to the expression trend of lncRNA H19, implying a possible negative regulatory relationship between lncRNA H19 and miR-148a (Fig. 4D). The expression of the remaining four miRNAs predicted in bioinformatics did not conform to the expected lncRNA H19 target expression changes in chondrocytes (data not shown). Therefore, miR-148a was finally identified as a possible direct target gene of lncRNA H19 in chondrocytes. Further, the results of dual-luciferase reporter assay further confirmed the interaction and binding sites between miR-148a and lncRNA H19 (Fig. S3). In addition, moderate intensity mechanical stress partially reversed IL-1β-induced changes in chondrocyte metabolism and inflammation from both the gene and protein expression levels (Fig. 4D and E).

Then, siRNA technology was performed to knock down the expression of lncRNA H19 in chondrocytes. The expression trend of miR-148a was found to be opposite to that of lncRNA H19 (as shown in Fig. 4G). Moreover, chondrocyte activity was significantly decreased in the si-H19 group compared to the Vector NC group (Fig. 4H). Immunofluorescence results showed that si-H19 significantly promoted the expression of MMP13 and significantly suppressed the expression of COL-II in chondrocytes (Fig. 4I). Importantly, we further found that si-H19 had no effect on chondrocyte pyroptosis (Fig. S1), although our animal results indicated the presence of chondrocyte pyroptosis in OA rat cartilage (3A-B), whereas there was a significant inhibition of chondrocyte autophagy (Fig. 4J–K). Briefly, si-H19 significantly suppressed the expression of LC3B, a marker of autophagy (Fig. 4J). The results of transmission electron microscopy also further showed that the autophagy level of chondrocytes in the si-H19 group was suppressed (Fig. 4K). The mitochondria (M) of chondrocytes in the si-H19 group were mostly obviously swollen, with localized lysis of the matrix within the membrane, and the cristae were reduced or disappeared. And the autolysosome (AP) and autophagosome-lysosome (ASS) were individually present. Most of the chondrocyte mitochondria (M) in the NC group were structurally normal with intact membranes and only some were slightly swollen. And the number of AP and ASS was relatively high. Transmission electron microscopy results indicated that si-H19 significantly inhibited chondrocyte autophagy. Further, we found that moderate-intensity mechanical stretching partially reversed the inhibitory effect of si-H19 on chondrocyte autophagy and promoted chondrocyte metabolic homeostasis (Fig. 4L–M).

## 4 Discussion

In our previous study, we demonstrated the mechanosensitivity of lncRNA H19 in a treadmill running-induced PTOA mouse model and verified the possible involvement of lncRNA H19 in the regulatory role of PTOA [44]. However, probably due to the relatively small duration and intensity of high-intensity treadmill exercise, only mild cartilage damage was caused in the knee joints of mice. Therefore, we look forward to further optimizing the treadmill running-induced PTOA model and further exploring the potential regulatory role of lncRNA H19 in this abnormal biomechanical factor-induced PTOA. It is well known that the main principle of surgically induced PTOA models, mainly including ACLT and destabilization of the medial meniscus (DMM), is wear and tear of the articular cartilage due to disruption of the normal structure of the joint resulting in excessive mechanical load. The ACLT model is one of the most widely used surgical models for PTOA, which can show similar features to human OA, including degeneration of articular cartilage and subchondral osteosclerosis [48]. Compared to DMM surgery, ACLT-induced PTOA is more susceptible to mechanical loading and is closer to the natural progression of PTOA in humans in a sports injury state, due to the fact that ACL injury is a common sports injury in the adolescent population. In addition, treadmill running is a good model for rodent PTOA, which can not only overcome the difficulties of rodent joint cavity surgery or injection operations, but also meet the needs of OA modeling or OA rehabilitation by controlling the intensity of treadmill running (high-intensity treadmill running can cause cartilage injury, while moderate-intensity treadmill running can delay cartilage injury). Therefore, in this study, ACLT combined with high-intensity treadmill exercise was used to mimic the pathogenesis of PTOA after sports injury, which is an optimized model of abnormal biomechanically induced PTOA. The efficiency of the optimized PTOA model was explored by detecting the pathology on rat cartilage and subchondral bone in three models (ACLT alone, treadmill running alone and, ACLT combined with treadmill running), while the role of mechanosensitive lncRNA H19 in PTOA was further explored.

Previous studies have shown that moderate intensity aerobic exercise can reduce joint loading by lowering body weight, thereby alleviating cartilage damage and reducing the risk of OA [49]. In the present study, we found that there was no significant weight loss in the treadmill running group rats (Fig. 1A). The range of changes in the mean body weight of the rats in the treadmill running group was within 30 g per week, which is within the range of changes in body weight during normal rat growth [50,51], suggesting that slight changes in body weight have very little effect on knee joint loading in the treadmill running group. In addition, it is believed that obese rats should exceed the normal rat body weight by more than 15 % for their knee joints to show a significant increase in load, which in turn has an effect on knee cartilage damage [52]. All rats were fed with conventional rat chow. The body weights of the rats in both the exercise and non-exercise groups were in the normal range, further indicating that the effect of body weight changes in the rats on PTOA was minimal in this experiment.

The subchondral bone is in a continuous dynamic bone remodeling process that relies on coupled bone formation and bone resorption to ensure the stability of the subchondral bone microstructure [53]. The occurrence of abnormal bone remodeling is considered to be a hallmark of OA. The early stage of OA is dominated by resorption of subchondral bone, as evidenced by reduced bone volume and possible stress microfractures, while the late stage of OA is dominated by formation of subchondral bone, as evidenced by increased bone volume and bone density, with significant subchondral bone sclerosis and osteophyte [54]. Considering the function of the ACL and the biomechanical properties of the ACL after injury, the medial and lateral tibial plateaus of the knee were reconstructed and analyzed separately after Micro-CT scanning to detect changes in the bone remodeling of the medial and lateral subchondral bone of the tibial plateau. As shown in Fig. 2, only the ACLT + R group had significantly higher trabecular thickness in the medial tibial plateau compared with the Sham and R groups, while the ACLT + R group had significant changes in all parameters of the trabeculae in the lateral tibial plateau, showing an increase in trabecular thickness and a decrease in the number of trabeculae. However, previous studies have noted that DMM-induced PTOA primarily leads to lesions in the medial tibial plateau [55]. A possible explanation for this discrepancy is that the DMM surgery damaged the medial meniscus and the medial collateral ligament, resulting in damage to the medial weight-bearing region of the joint due to abnormal mechanical load, whereas the lateral femoral condyle and the anteroposterior tibia are more prone to impingement after ACL rupture, resulting in significant lesions in the lateral region of the knee. In addition, the high-intensity treadmill running further aggravated the impingement within the knee joint. Excessive mechanical stimulation inhibits the function of bone surface osteoblasts and activates osteoclasts, resulting in a decrease in the number of local trabeculae and an increase in trabecular space [56]. Moreover, it has to be acknowledged that the relatively small sample of our micro-CT scans (n = 5) may also be a possible reason for the absence of significant differences in the morphological indexes of the lateral tibial plateau bone. Further evidence is needed to verify that ACLT-induced PTOA is more severely damaged laterally, whereas DMM-induced PTOA is more severely damaged medially.

Articular cartilage is a smooth, elastic, and dense connective tissue that reduces bone-to-bone friction during joint movement. Prolonged mechanical load or frequent, repetitive, high-intensity exercise can lead to wear and tear of articular cartilage. The lack of blood supply to the articular cartilage makes it difficult to regenerate, so the wear and tear of cartilage due to damage to intra-articular structures is a major risk factor for the onset and progression of PTOA, which is a significant pathological feature of PTOA. In this study, the status of articular cartilage damage and calcification in rats was evaluated by HE staining and red solid green staining combined with Mankin's and OARSI scores (Fig. 1C and D). The results showed that Mankin's score and OARSI score were significantly higher in the ACLT group, R group, and ACLT + R group, compared to the Sham group. The degree of cartilage damage was in descending order for the ACLT + R group, ACLT group, and R group, indicating that ACLT combined with treadmill running could exacerbate surgery-induced or treadmill running-induced articular cartilage damage alone. Therefore, ACLT combined with treadmill running for PTOA rat modeling method is superior to ACLT alone. Further, immunohistochemical staining and WB results showed (Fig. 1, Fig. 3B) that ACLT surgery combined with treadmill exercise resulted in decreased COL-II expression and increased MMP-13 expression in cartilage tissue, suggesting destruction and degeneration of articular cartilage. Meanwhile, RT-qPCR results (Fig. 3A) also supported that cartilage catabolism was significantly enhanced in the ACLT group relative to the Sham group. Cartilage matrix degradation was more severe in the ACLT + R group than in the ACLT group, while no significant changes were observed in the R group. This suggests that treadmill running after ACLT surgery exacerbates injury-induced cartilage degradation, which is a promising animal model of PTOA induced by mechanical stress factors and could provide a basis for subsequent studies on the pathogenesis of biomechanical factors in OA. However, high-intensity treadmill running alone caused only minor cartilage degeneration, probably because there was no primary joint damage to the articular cartilage in the rats. And treadmill running does not involve high impact forces as well as rapid directional changes, which are important factors contributing to cartilage damage.

Pyroptosis is a Caspases-dependent pattern of programmed inflammatory cell death. Studies have shown that chondrocyte pyroptosis causes a variety of pathological changes including cartilage degradation and inflammatory responses. Inhibition of chondrocyte pyroptosis can alleviate cartilage damage and prevent the progression of OA disease [[57], [58], [59]]. Currently, Caspase-1-mediated classic pyroptosis has received much attention in OA. Stimulation by a variety of endogenous or exogenous factors such as LPS, ROS, bacteria, K^+^ efflux, Ca^2+^ endocytosis, lysosomal destabilization, mitochondrial damage, and cell swelling can initiate the assembly of inflammasome to cleave Caspase-1 precursors and release activated Caspase-1. Subsequently, activated caspase-1 cleaves Gasdermin D (GSDMD) and releases the N-terminal structural domain and transports it to the plasma membrane to oligomerize into a 10–15 nm diameter pore, thereby releasing pro-inflammatory cytokines such as IL-1β and IL-18 into the extracellular environment and ultimately inducing pyroptosis [60,61]. NLRP3 is an important regulator of the inflammatory response and cell death. Activation of NLRP3 inflammasome triggers the release of pro-inflammatory cytokines to induce chondrocyte pyroptosis [62,63], thus participating in the initiation and development of OA [64]. The results of RT-qPCR and WB (Fig. 3A and B) showed that the expression levels of both inflammatory factors and pyroptosis-related factors were significantly upregulated in the ACLT + R group (p < 0.01), indicating that ACLT surgery combined with treadmill running could induce pyroptosis in rat cartilage. However, unfortunately, in our experiments, knockdown of lncRNA H19 in chondrocytes resulted in no significant changes in pyroptosis-related genes and protein (Fig. S1).

Autophagy is a self-degrading process that is important for the homeostasis of bone and cartilage. Autophagy can promote cell survival by adapting cells to stress conditions [11]. Recent studies revealed that autophagy may be closely related to the initiation and development of OA [65]. It has been shown that decreased expression of autophagy markers correlates with proteoglycan loss in cartilage of osteoarthritic mice [66]. Studies have also shown that some autophagy-inducing agents have a protective effect on OA [14,15]. In addition, Wang et al. [67] demonstrated lncRNA H19 can promote glioblastoma multiforme development by activating autophagy. Therefore, we propose the hypothesis that lncRNA H19 may be involved in the regulation of PTOA through activation of autophagy. We further detected the level of autophagy-related genes and proteins after knocking down lncRNA H19 in chondrocytes. The results showed that knockdown of lncRNA H19 significantly suppressed autophagy levels in primary chondrocytes, accompanied by disturbed chondrocyte metabolism. Transmission electron microscopy results similarly showed that the autophagy level of chondrocytes in the si-H19 group was inhibited, whereas the autophagy level of most chondrocytes in the NC group was normal. Further studies have found that moderate-intensity mechanical stretching partially reversed the inhibitory effect of si-H19 on chondrocyte autophagy and promoted chondrocyte metabolic homeostasis. This suggests that moderate-intensity mechanical stimulation may activate chondrocyte autophagy levels by upregulating the expression of lncRNA H19, which in turn alleviates alleviate cartilage damage.

The results of our animal experiments showed that lncRNA H19 showed significantly low expression in the articular cartilage of rats in the R group and ACLT + R group, suggesting that lncRNA H19 is mechanosensitive and may be involved in regulating the pathogenesis of exercise-induced PTOA in rats. Accumulating evidence suggests that lncRNAs can indirectly regulate the function of downstream mRNAs by targeting miRNAs, thereby affecting cell proliferation and differentiation [68]. Therefore, our bioinformatic analysis of the target genes of lncRNA H19 combined with RT-qPCR validation identified miR-148a as its possible direct target (Fig. 4C and 4D). It has been demonstrated that lncRNA-H19 can regulate ischemic stroke induced oxidative stress damage by increasing the expression of Rho-associated protein kinase 2 (Rock2) through the regulation of miR-148a, thus exerting a neuroprotective effect [69]. Similarly, Wu [70] et al. showed that lncRNA H19 can promote the progression of laryngeal squamous cell carcinoma (LSCC) by targeting miR-148a and DNA methyltransferase DNMT1. All of these results demonstrate that lncRNA H19 can directly target miR-148a for functional regulation. In addition, miR-148a has been shown to be elevated in the plasma and bone marrow of patients with osteoporosis [71,72]. Another study reported that miR-148a can block osteoblast differentiation and bone remodeling by disrupting p300-dependent nuclear factor E2-related factor 2 (Nrf 2) pathway activation [73], suggesting that miR-148a plays an important role in bone metabolic diseases. To investigate the regulatory role of lncRNA H19/miR148a axis in OA chondrocyte metabolism, IL-1β was used to induce chondrocyte OA-like alterations *in vitro*. Subsequently, moderate intensity mechanical tension force was loaded onto the primary chondrocytes. It was found that moderate-intensity mechanical tension stress partially reversed the IL-1β-induced decrease in lncRNA H19 expression, while the upregulation of miR-148a expression was also partially reversed, suggesting that the expression of lncRNA H19 and miR-148a may be negatively regulated in chondrocytes. To further verify the targeting relationship between lncRNA H19 and miR-148a and their regulatory mechanisms on OA chondrocyte metabolism, cell transfection techniques were used to knock down the expression of lncRNA H19. We found that the knockdown of lncRNA H19 was able to upregulate miR-148a expression, inhibit chondrocyte anabolism, and enhance chondrocyte catabolism and inflammatory response, suggesting that knockdown of lncRNA H19 promoted chondrocyte injury by targeting miR-148a.

## 5 Limitations

We have to admit that this study still has some limitations. For example, we only detected changes in lncRNA H19 expression in cartilage in vivo, without knocking down or repressing lncRNA H19 expression in vivo. Future studies are needed to validate the regulatory role of mechanosensitive lncRNA H19 in OA by combining gene knockdown or specific inhibitors to suppress the expression of lncRNA H19 in cartilage. In addition, the regulatory role of lncRNA H19/miR-148a/autophagy axis in chondrocytes needs further validation. Further investigation can be conducted to determine whether knocking down miR-148a or activating autophagy can partially reverse chondrocyte damage caused by knocking down lncRNA H19. Furthermore, it is worth mentioning that chondrocytes, as the only cell type, accounted for only 1 % of the cartilage tissue [74], suggesting that extracting high quality RNA/protein from rat cartilage tissue is difficult. In this study, the amount of protein in rat cartilage tissue remained low despite the use of a dedicated protein extraction kit, which resulted in a lack of quality in blots. Future studies could further explore how to extract high quality RNA/protein from cartilage tissue.

## 6 Conclusions

ACLT surgery combined with high-intensity treadmill running can successfully induce PTOA in rats, which may be a good rodent model to explore the pathological mechanism of PTOA influenced by biomechanical factors. In addition, we found that moderate-intensity mechanical tension stress increased the expression of lncRNA H19, decreased the expression of miR-148a, promoted chondrocyte anabolism, inhibited catabolism, and attenuated the inflammatory response, thereby alleviating IL-1β-induced chondrocyte injury. Knockdown of lncRNA H19 upregulates miR-148a expression and inhibits chondrocyte autophagy but not pyroptosis. In summary, this study demonstrated that mechanosensitive lncRNA H19 could target miR-148a and activate the autophagy level of chondrocytes, which in turn delayed chondrocyte degeneration and alleviated the development of PTOA. Moderate-intensity mechanical stress may alleviate cartilage damage through the lncRNA H19/miR-148a/autophagy axis. In the future, clinical trials can be considered to evaluate whether mechanosensitive lncRNA H19 may serve as a potential therapeutic strategy for PTOA.

## Funding

This work is supported by Key Laboratory of Sports and Physical Health of the Ministry of Education (Beijing Sport University), the Fundamental Research Funds for the Central Universities (20221021), and the Plan on enhancing scientific research in 10.13039/100009659GMU.

## Institutional review board statement

Approval has been given by the Ethical Committee of Beijing Sport University on the Care and Use of Animal Subjects in Research (Approval Number: 2023026A). All rats were anesthetized before the extraction of knee cartilage and were humanely euthanized after the procedures were completed.

## Informed consent statement

Not applicable.

## Data availability statement

The data presented in this study are also available from the corresponding author and first author upon request.

## CRediT authorship contribution statement

**Xuchang Zhou:** Writing – original draft, Funding acquisition, Conceptualization. **Hong Cao:** Writing – review & editing, Writing – original draft. **Tao Liao:** Software, Methodology. **Weizhong Hua:** Validation. **Ruobing Zhao:** Software. **Dongxue Wang:** Visualization, Validation. **Huili Deng:** Formal analysis, Data curation. **Yajing Yang:** Validation, Data curation. **ShengYao Liu:** Funding acquisition. **Guoxin Ni:** Funding acquisition, Conceptualization.

## Declaration of competing interest

The authors declare no conflict of interest.
