# Supplementary material for: Mechanosensitive lncRNA H19 promotes chondrocyte autophagy, but not pyroptosis, by targeting miR-148a in post-traumatic osteoarthritis
Source: Noncoding RNA Res. 2024 Jul 31;10:163–76. doi: 10.1016/j.ncrna.2024.07.005 (PMC11470567; doi:10.1016/j.ncrna.2024.07.005)

Figure 4L

COL-II

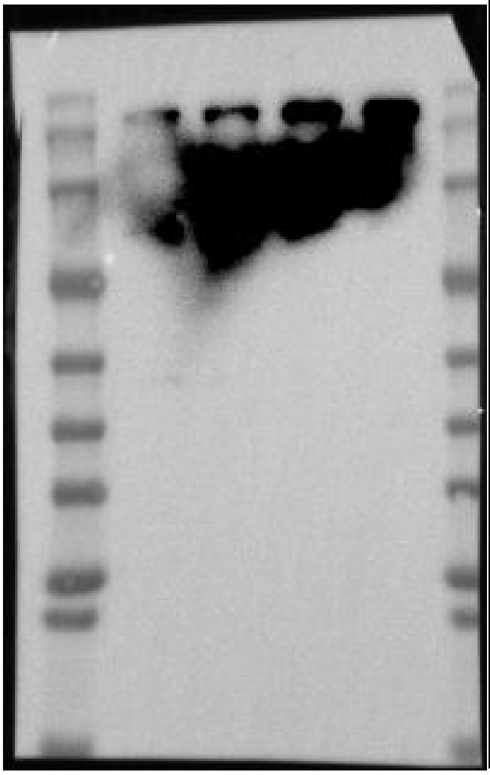

GAPDH

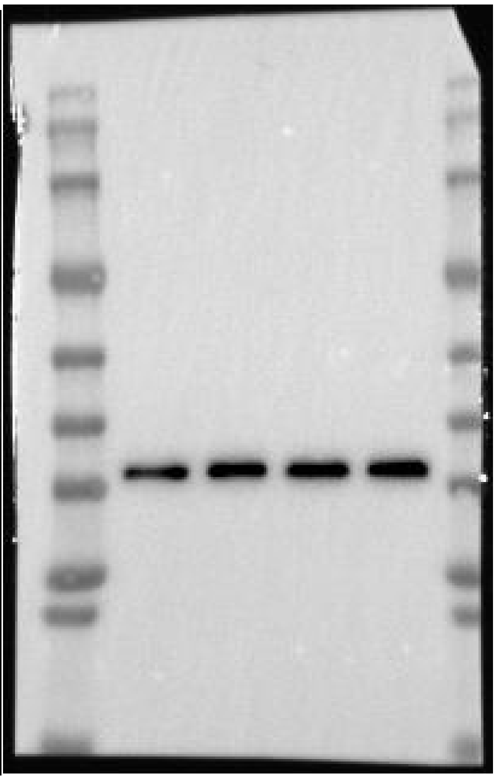

COL-II

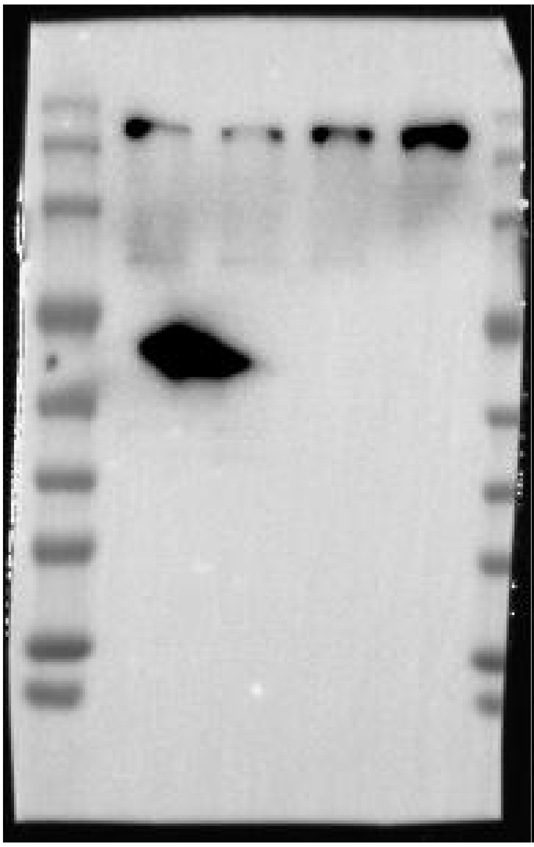

GAPDH

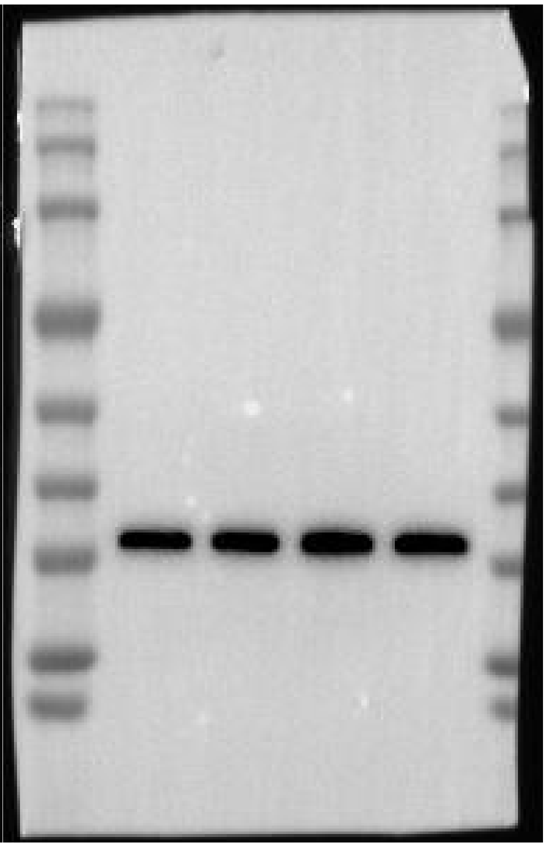

COL-II

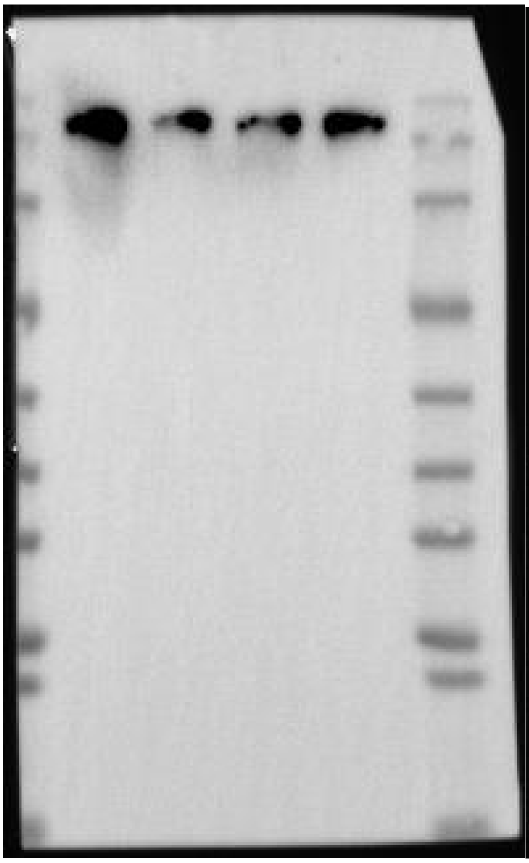

GAPDH

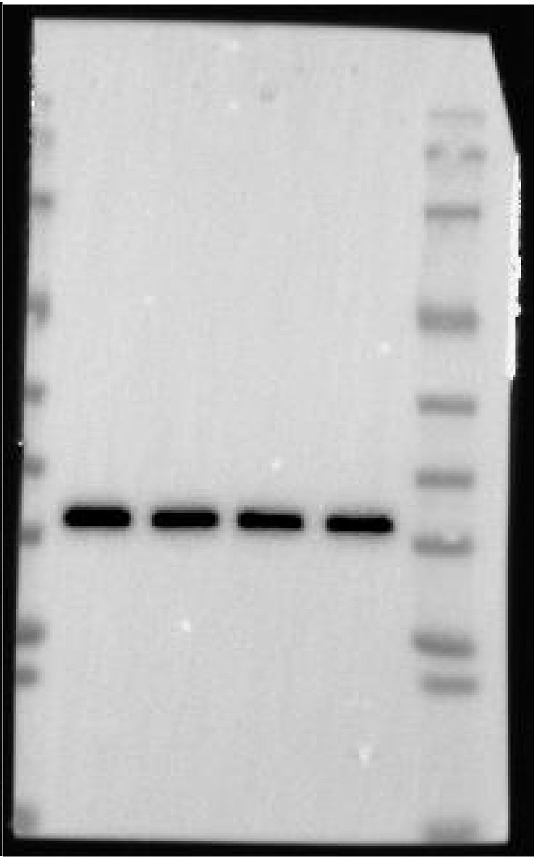

COL-II

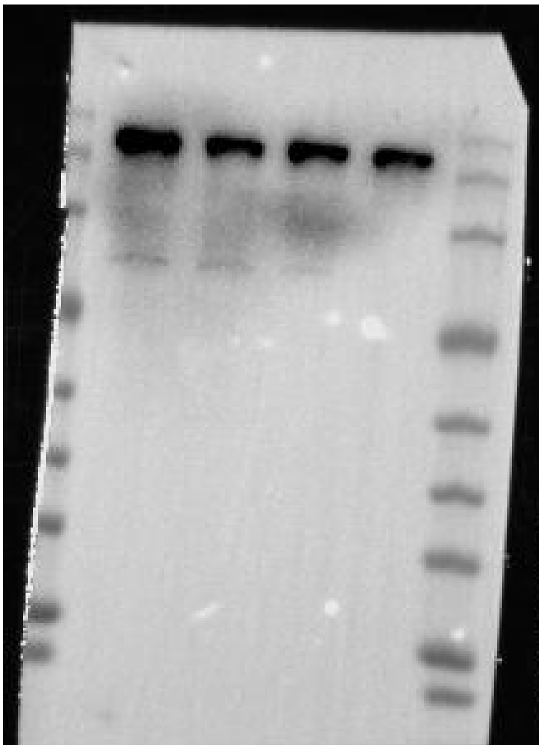

GAPDH

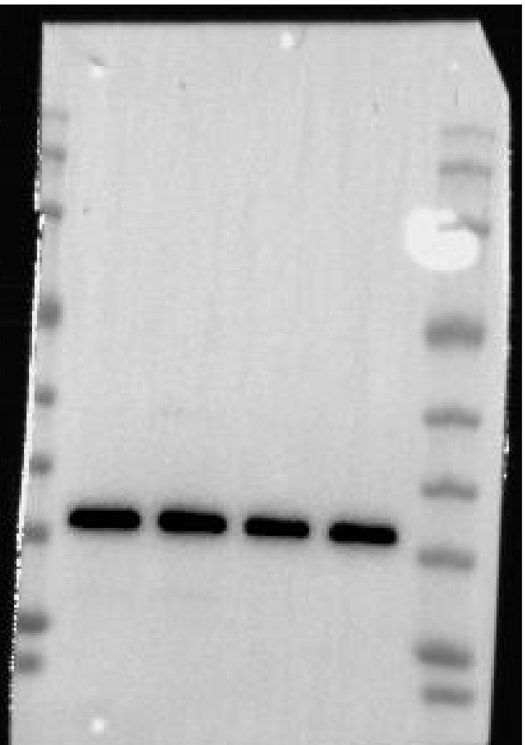

MMP13

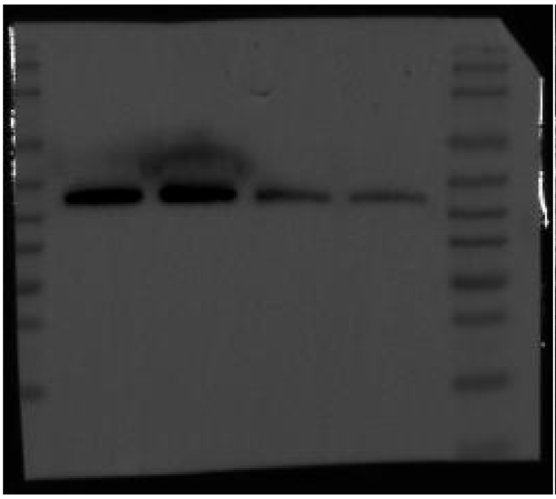

GAPDH

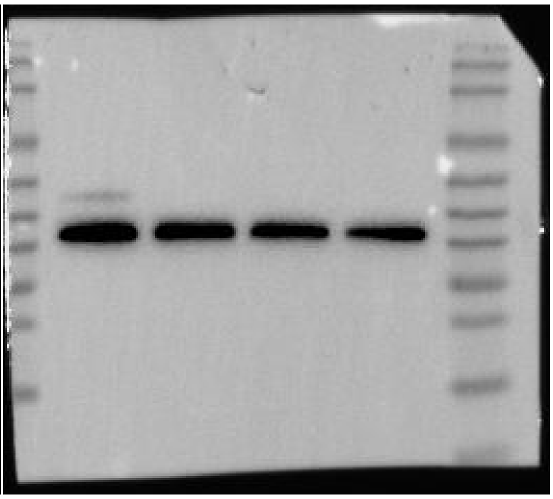

MMP13

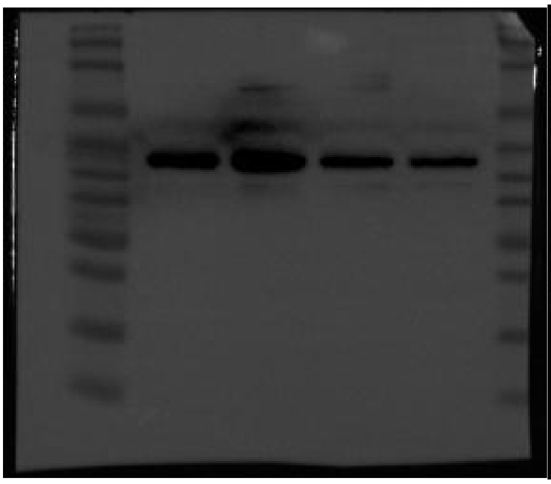

GAPDH

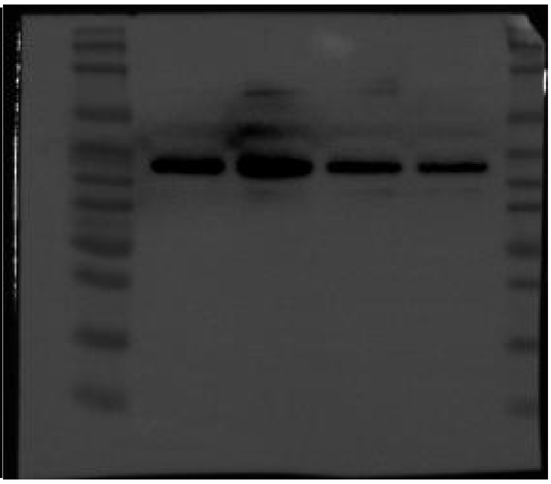

MMP13

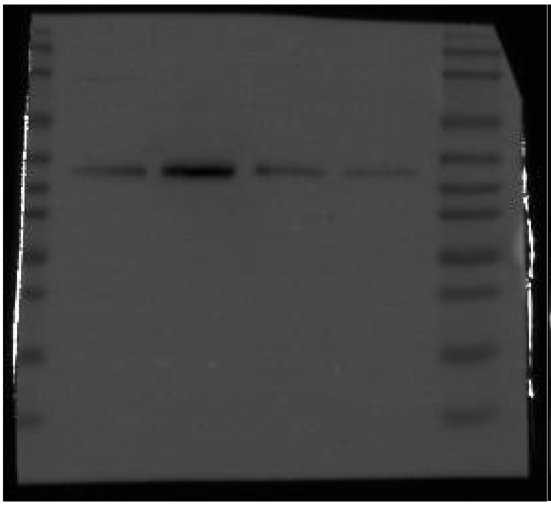

GAPDH

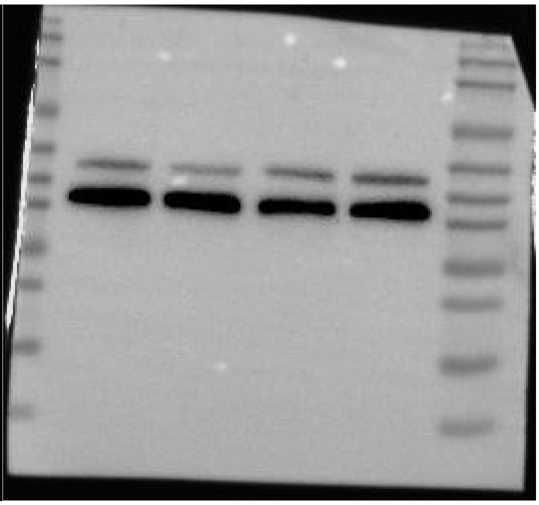

MMP13

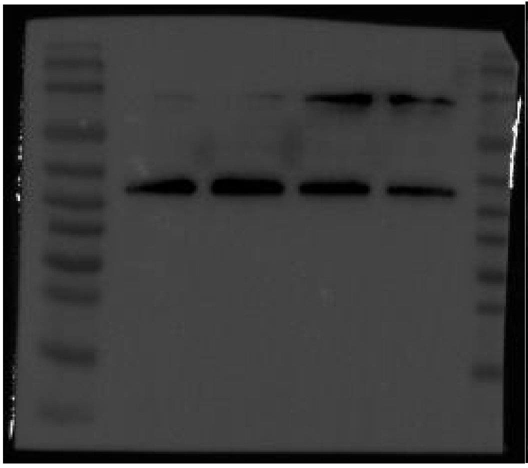

GAPDH

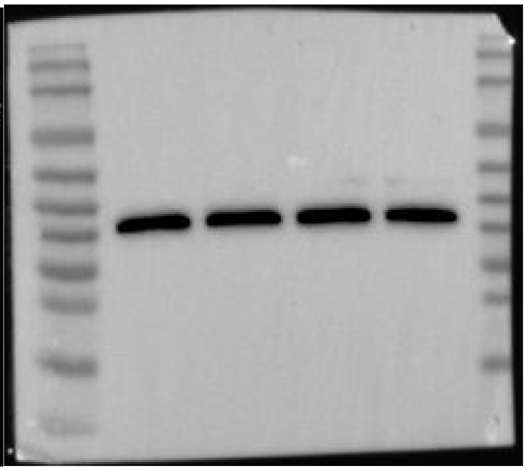

ACAN

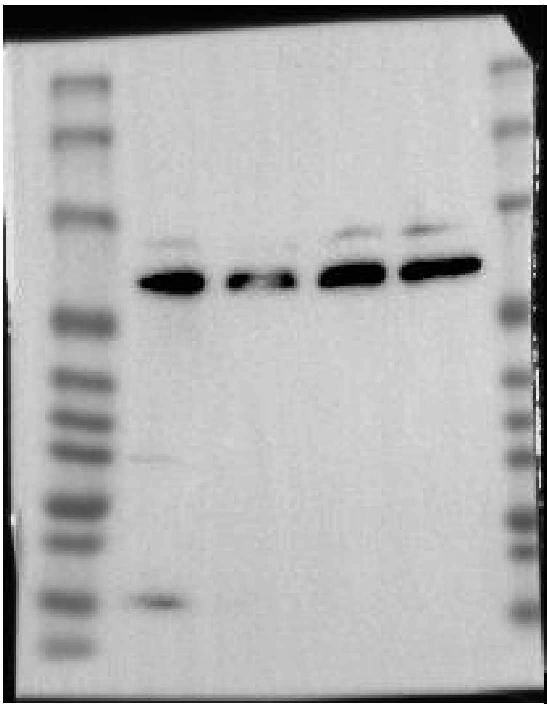

GAPDH

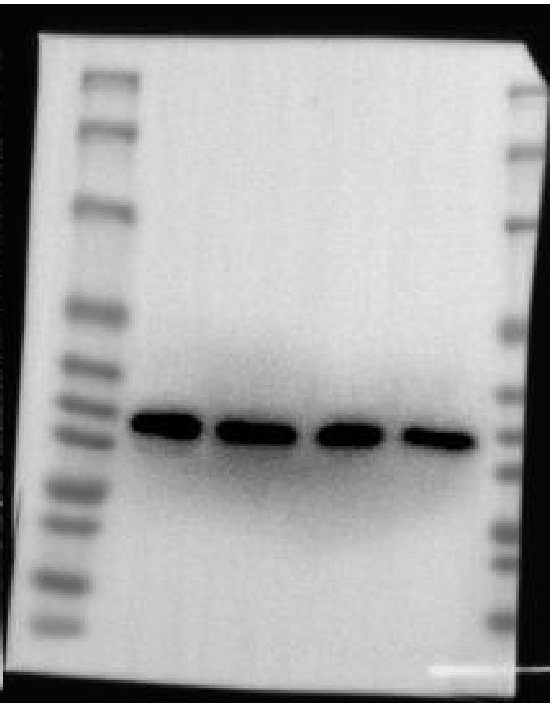

ACAN GAPDH

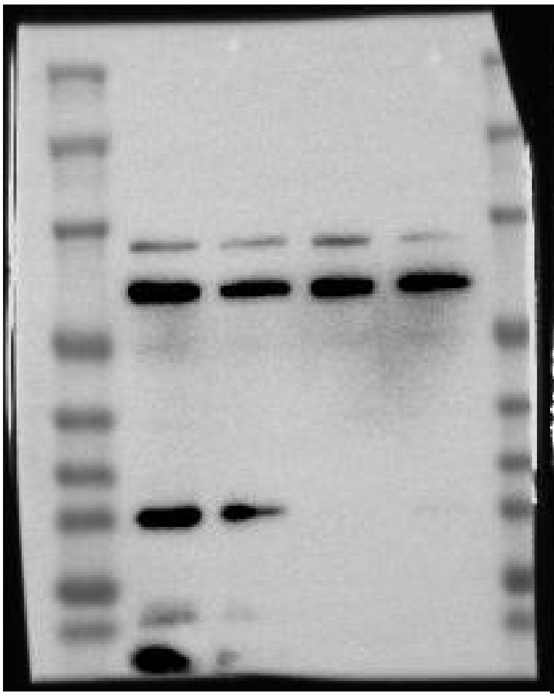

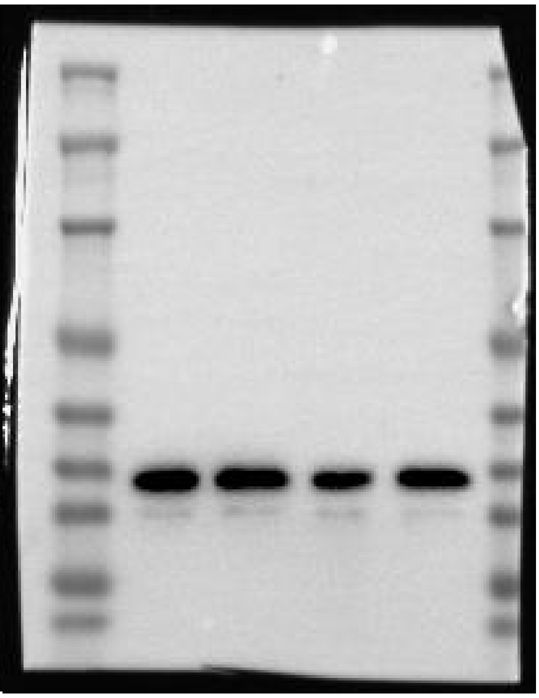

ACAN GAPDH

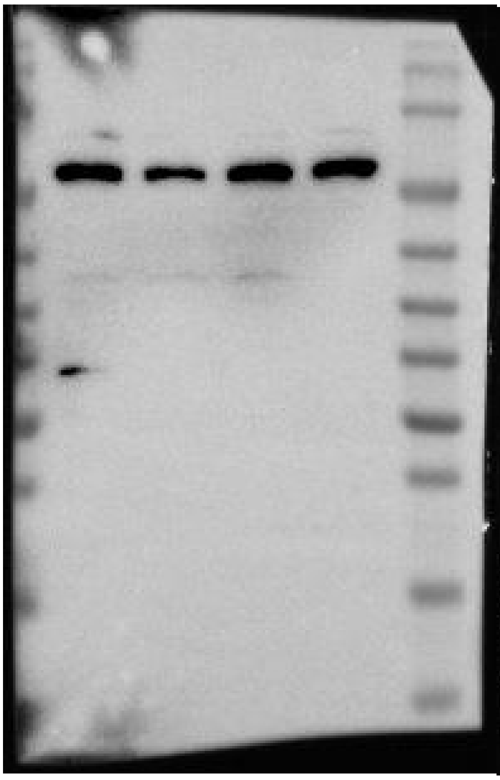

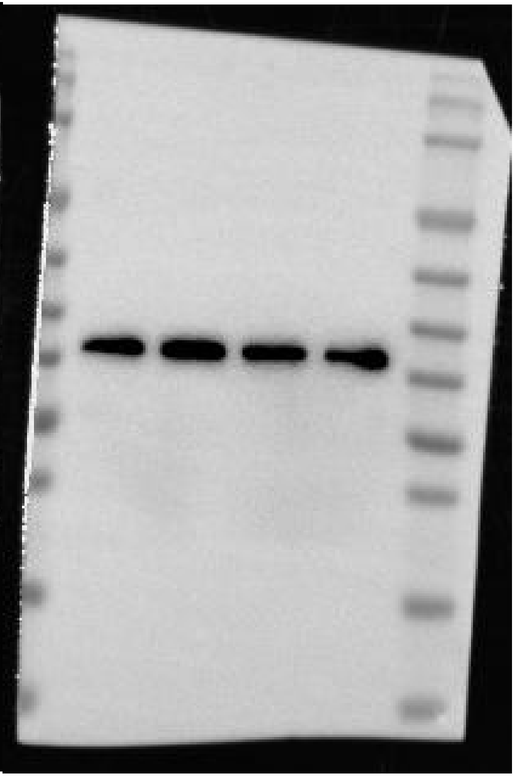

ACAN

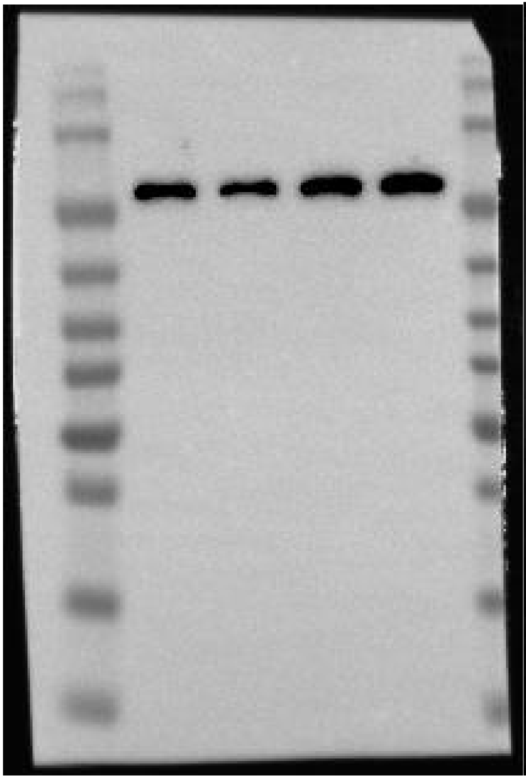

GAPDH

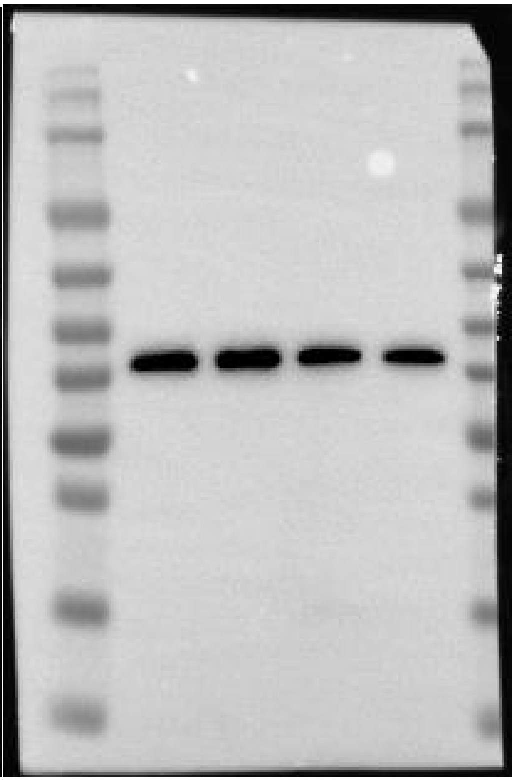

P62

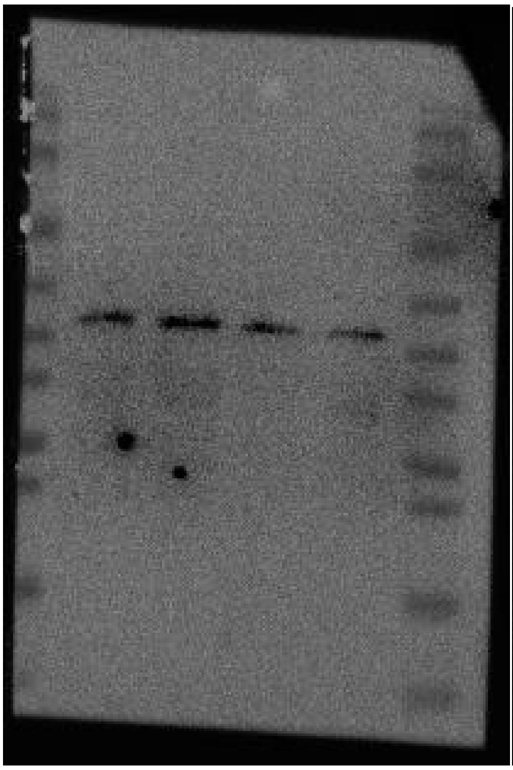

p62

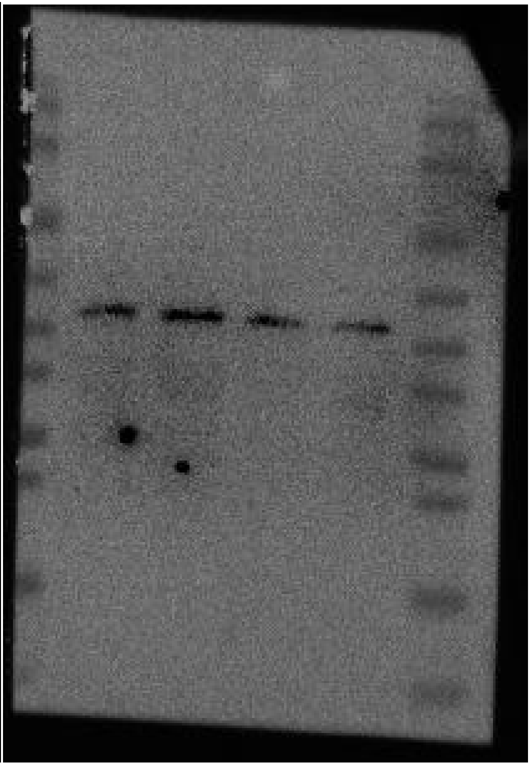

P62

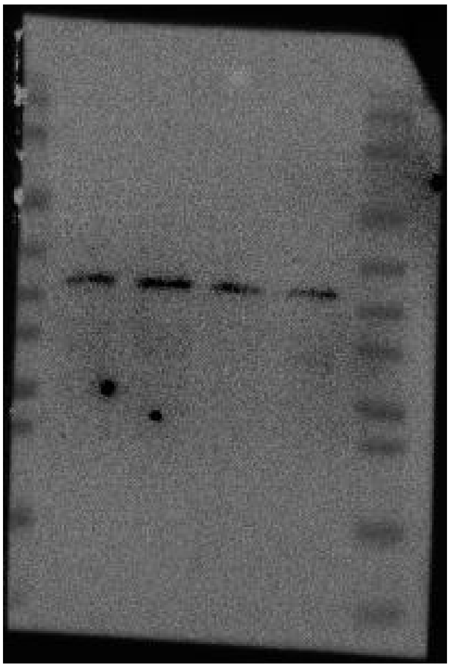

LC3B

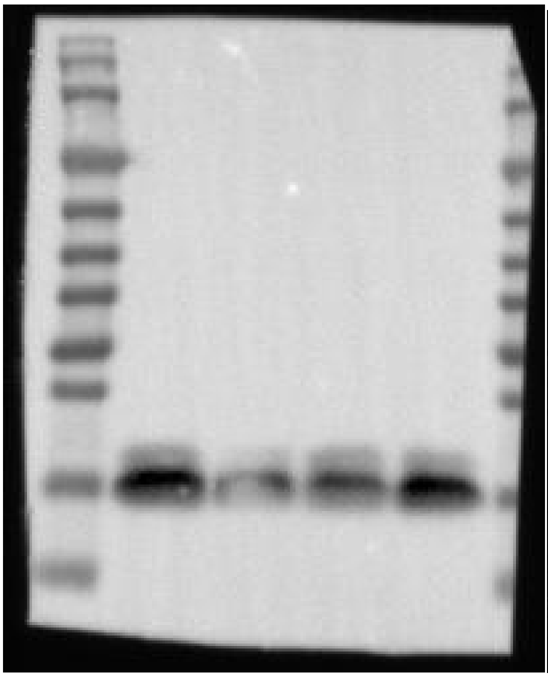

GAPDH

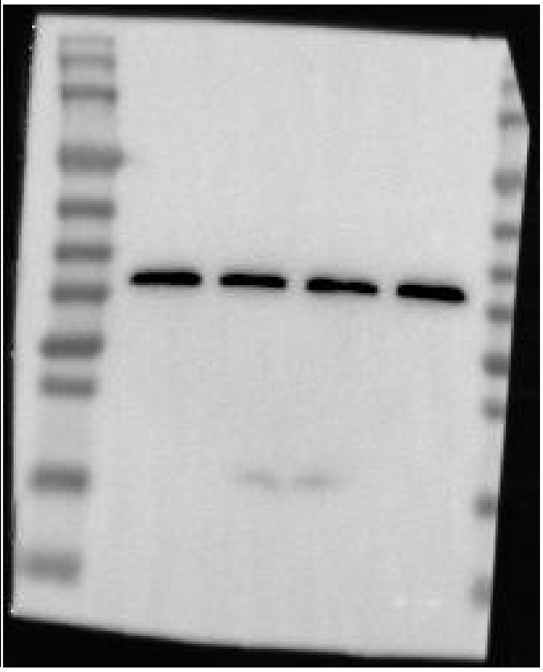

LC3B

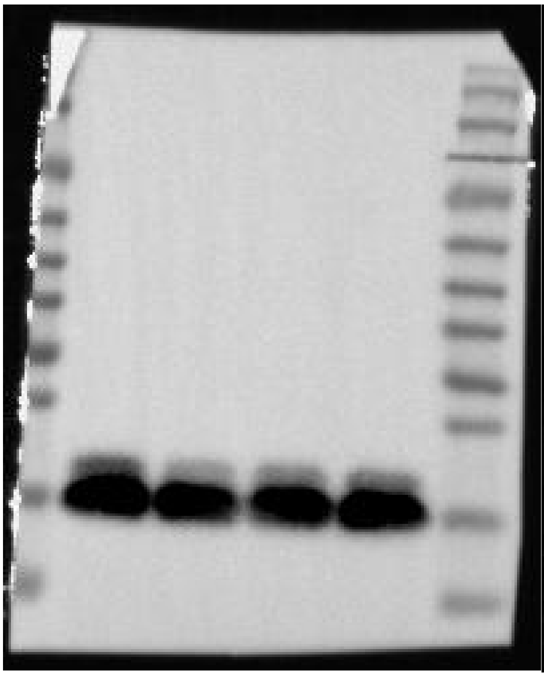

GAPDH

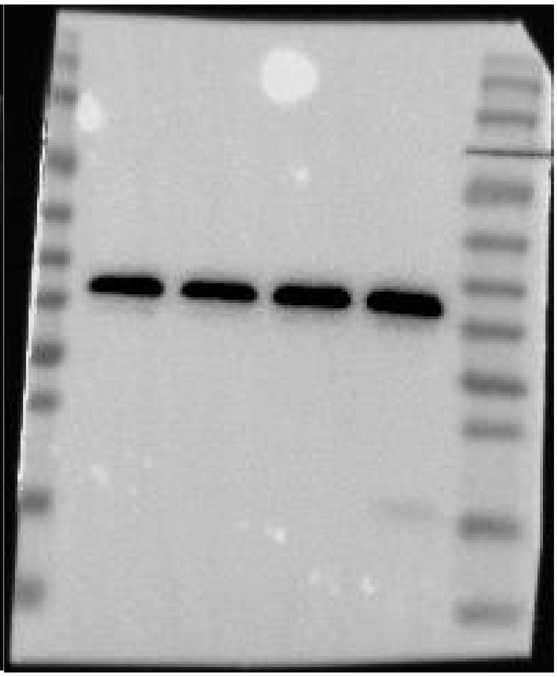

LC3B

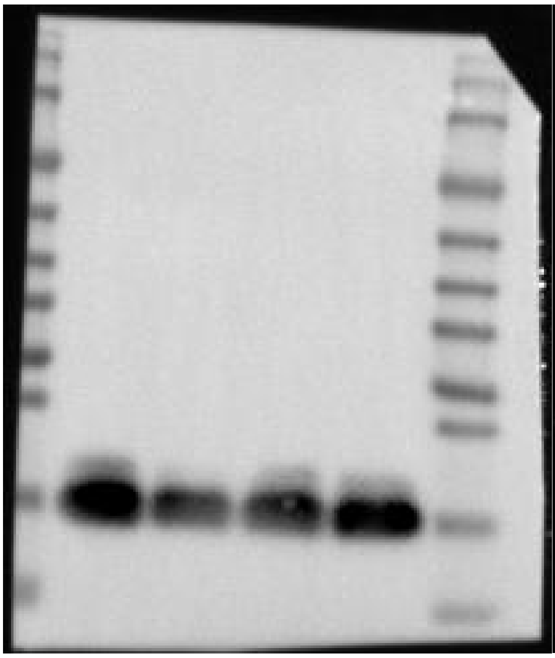

GAPDH

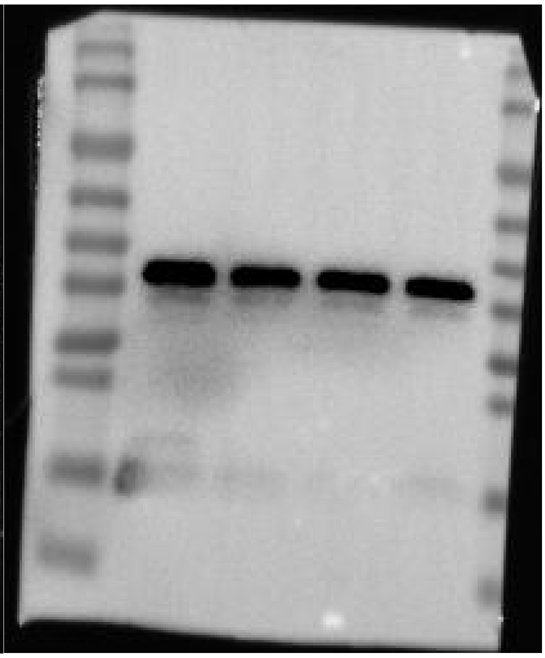

LC3B

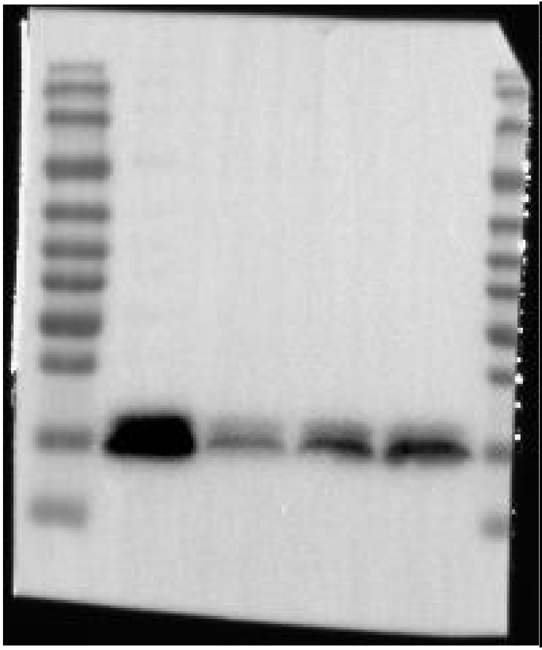

GAPDH

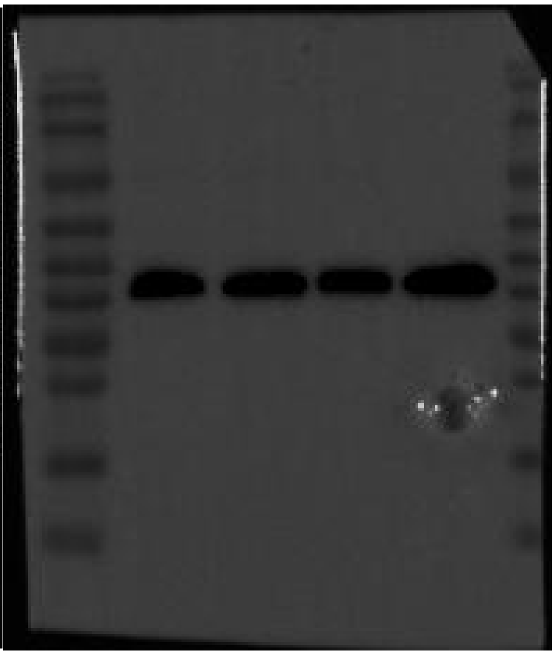

LC3B

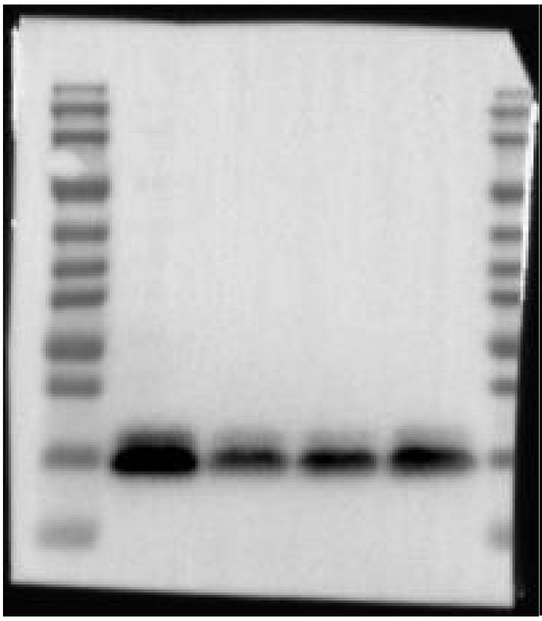

GAPDH

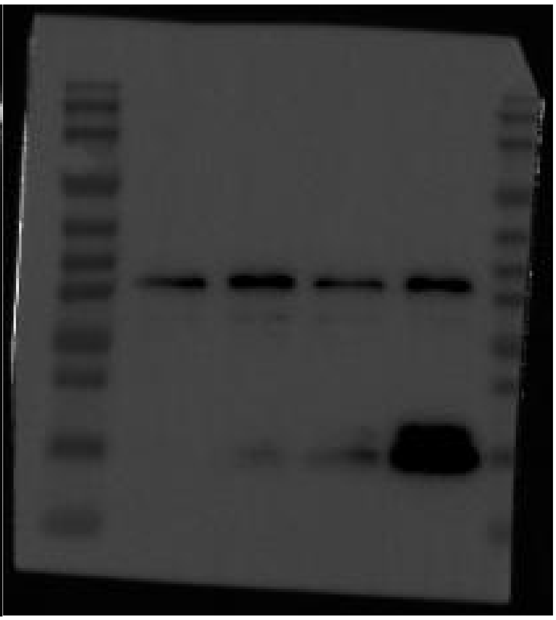

LC3B

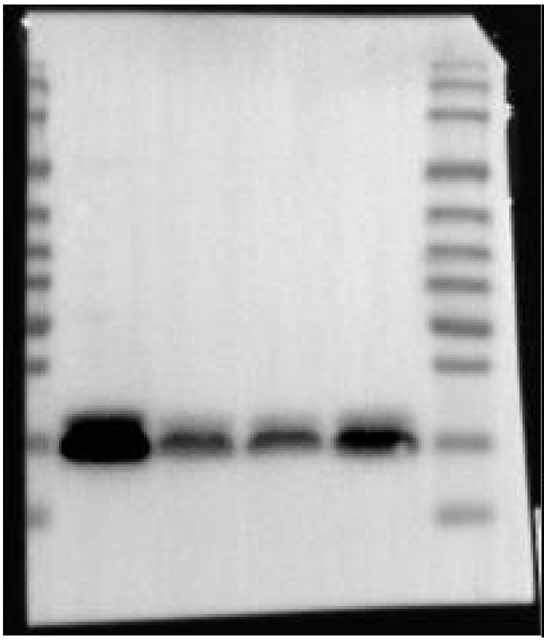

GAPDH

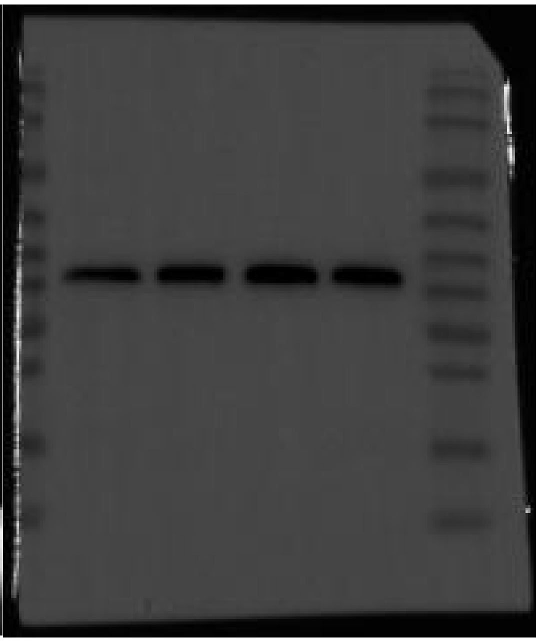

LC3B

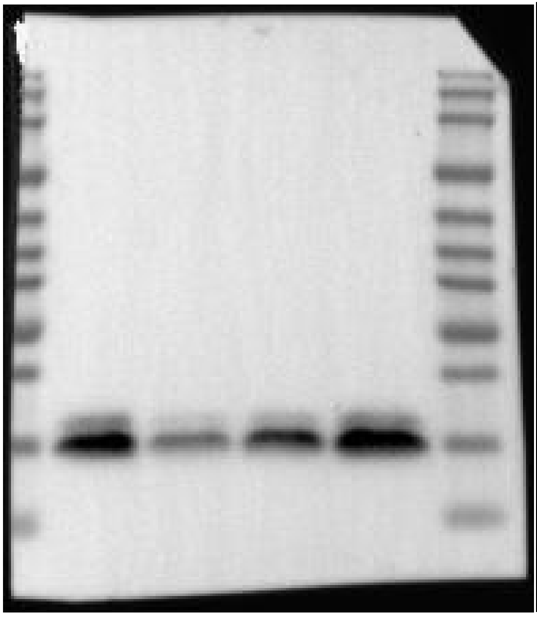

GAPDH

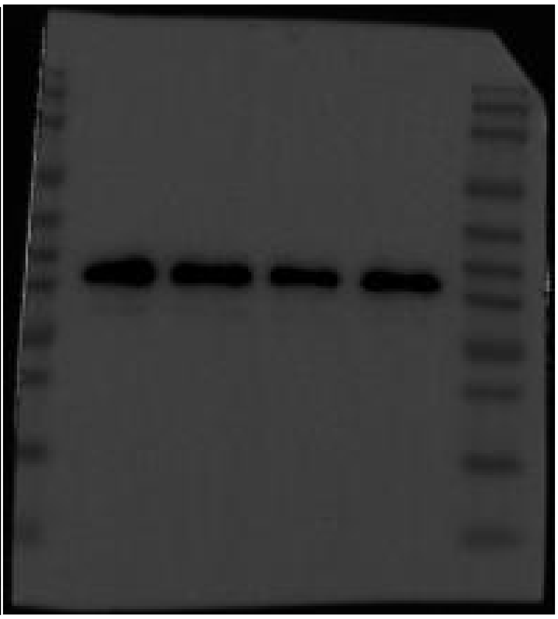

Figure 4E

ACAN

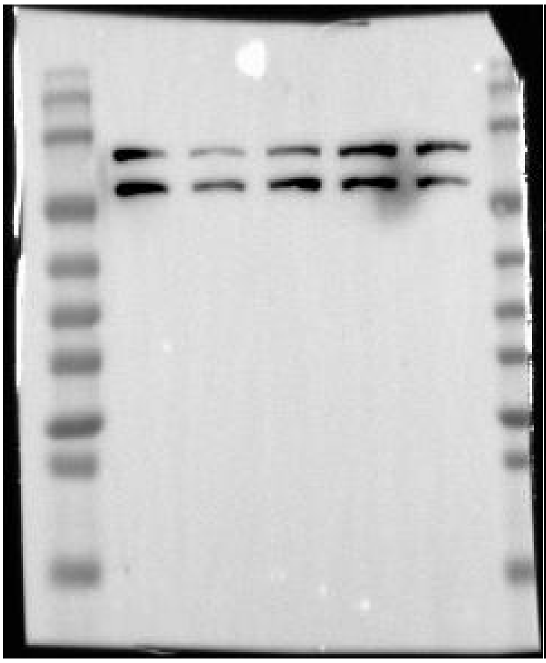

GAPDH

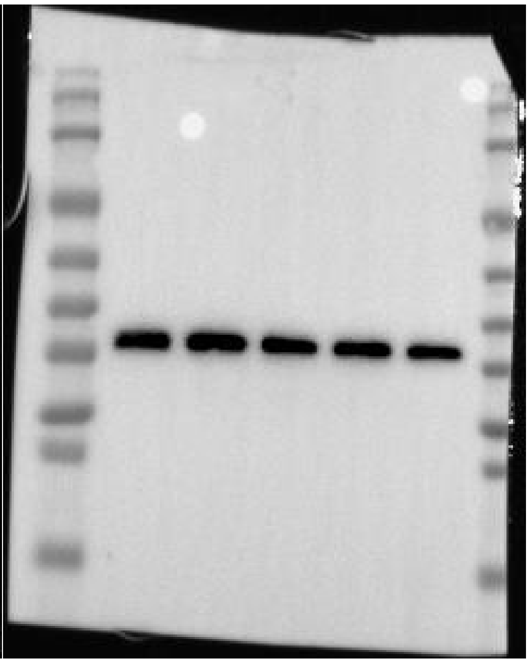

ACAN

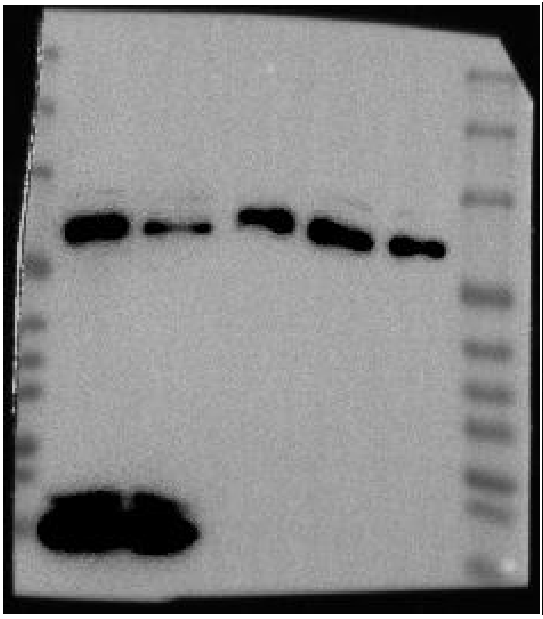

GAPDH

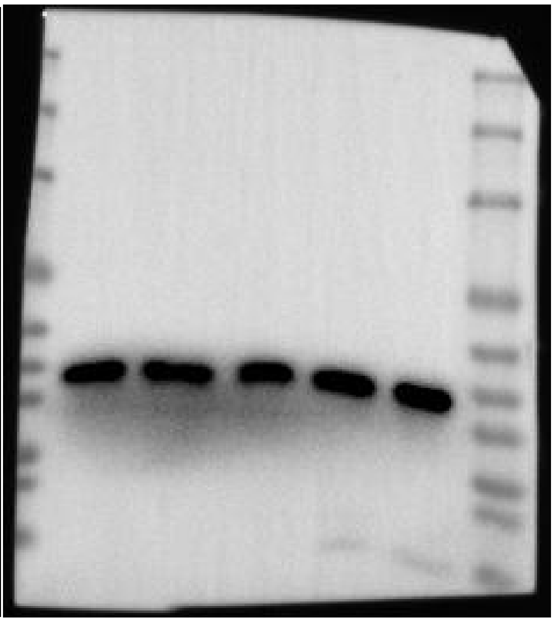

ACAN

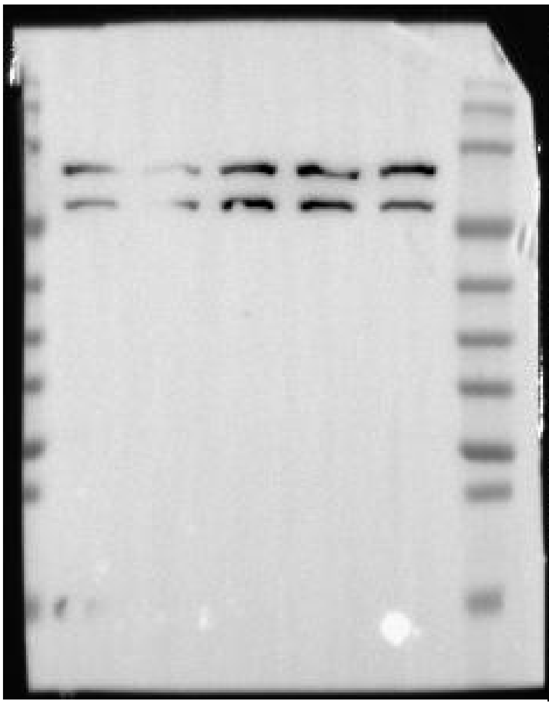

GAPDH

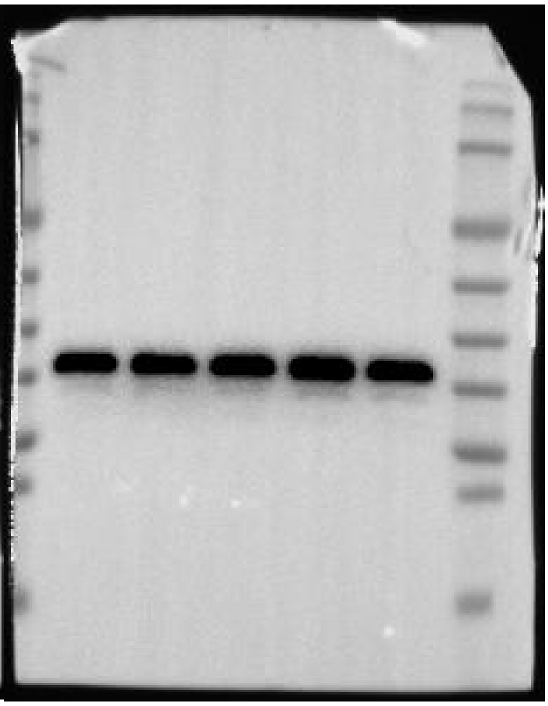

ACAN

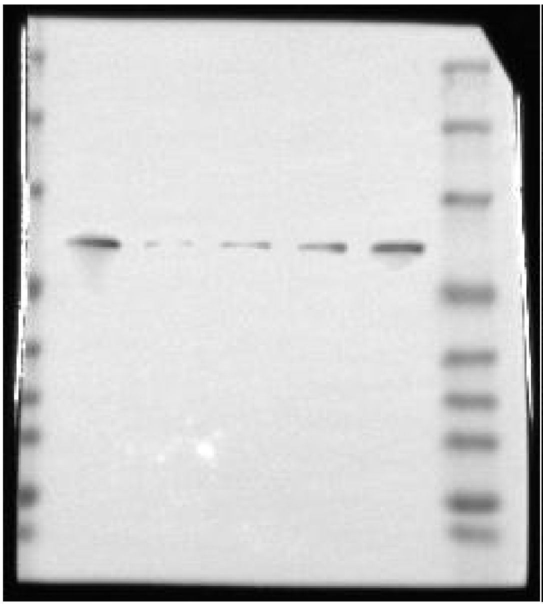

GAPDH

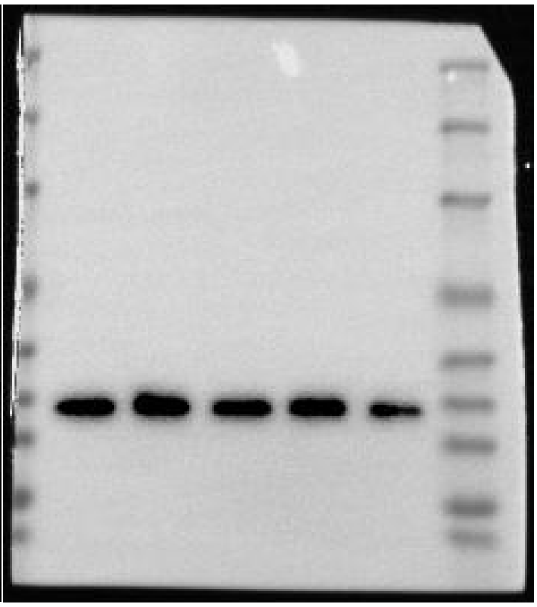

COL-II

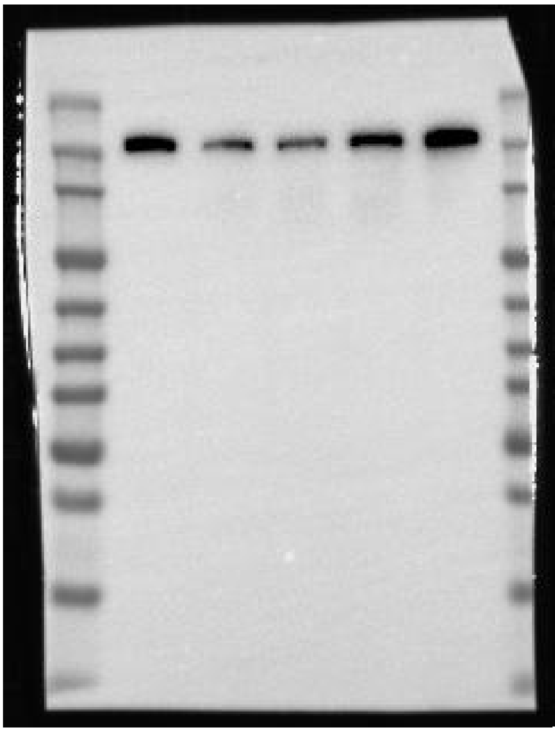

GAPDH

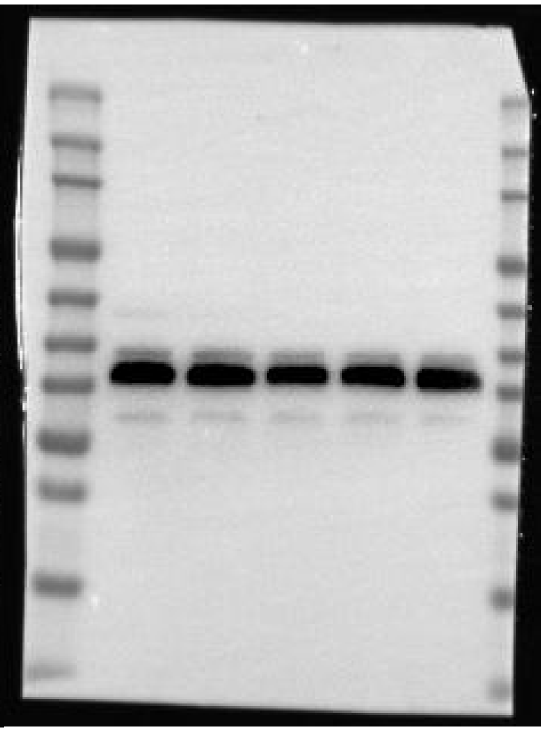

COL-II

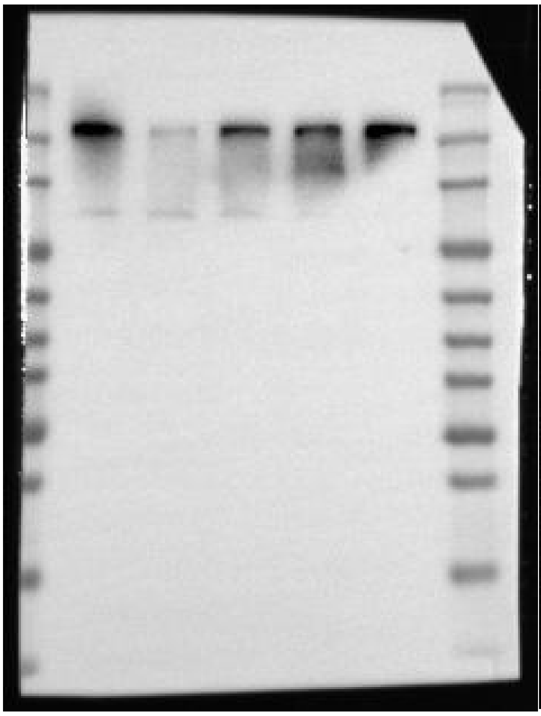

GAPDH

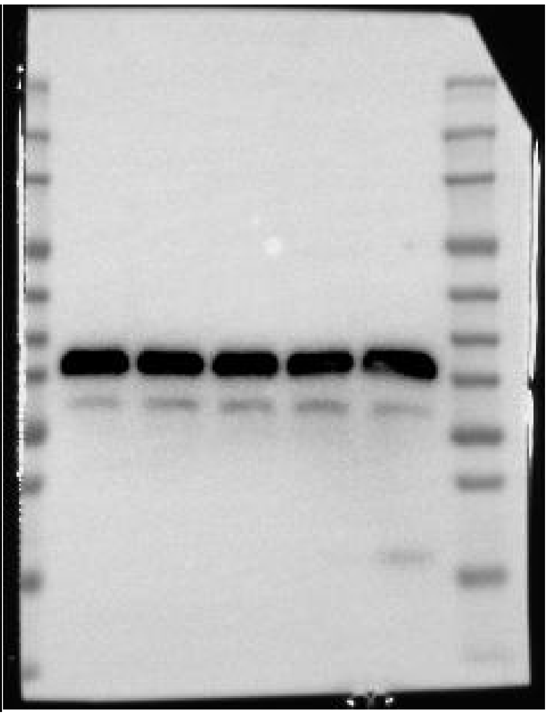

COL-II

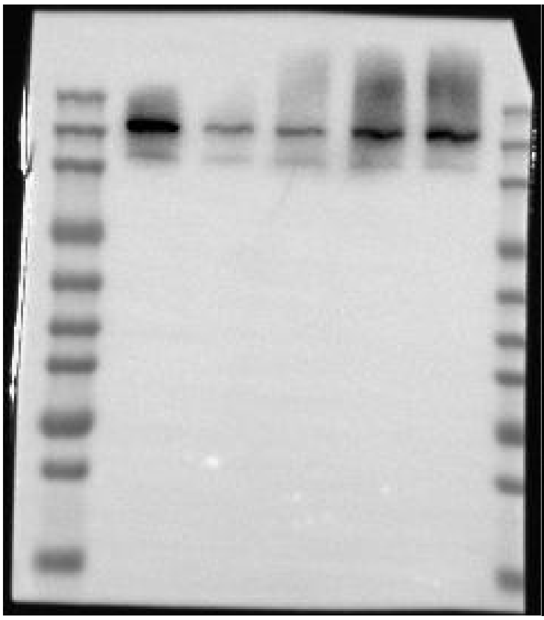

GAPDH

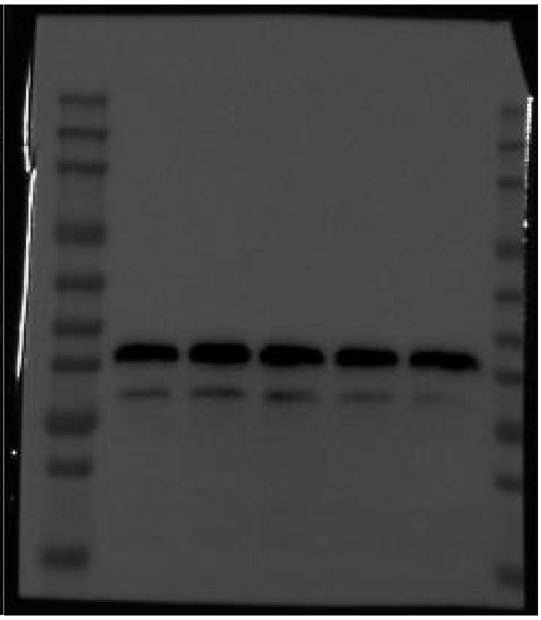

COL-II

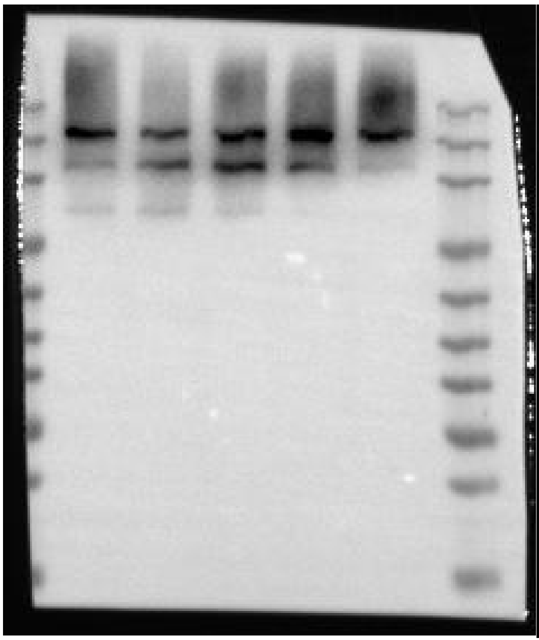

GAPDH

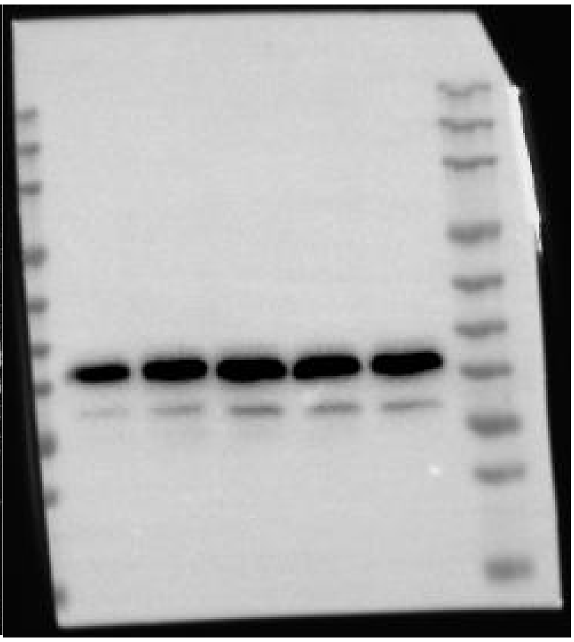

MMP13

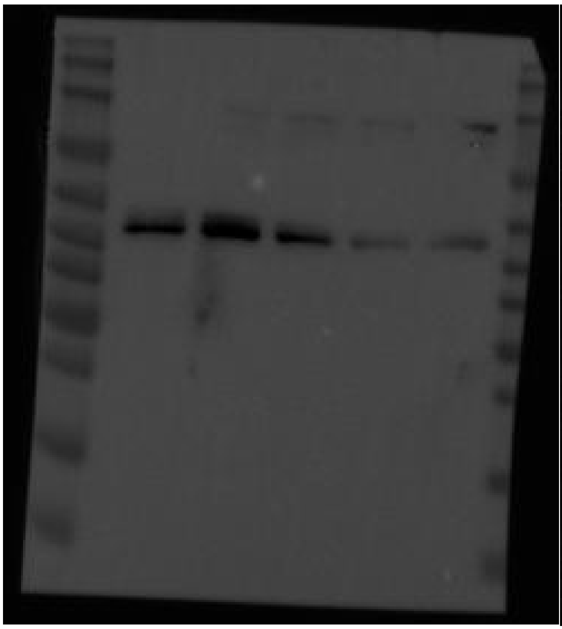

GAPDH

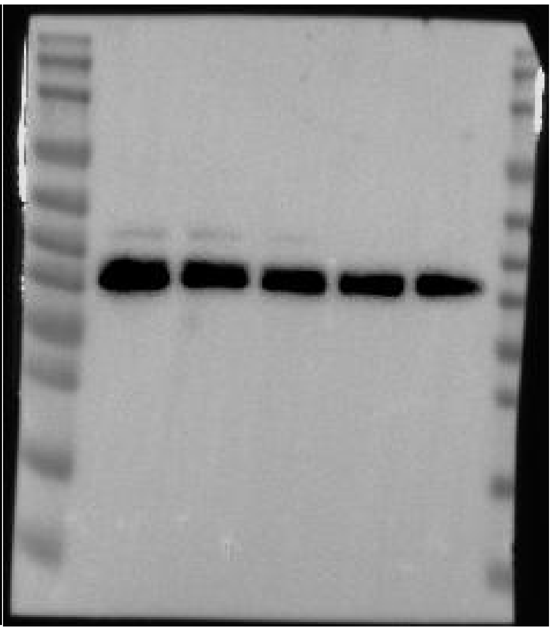

MMP13

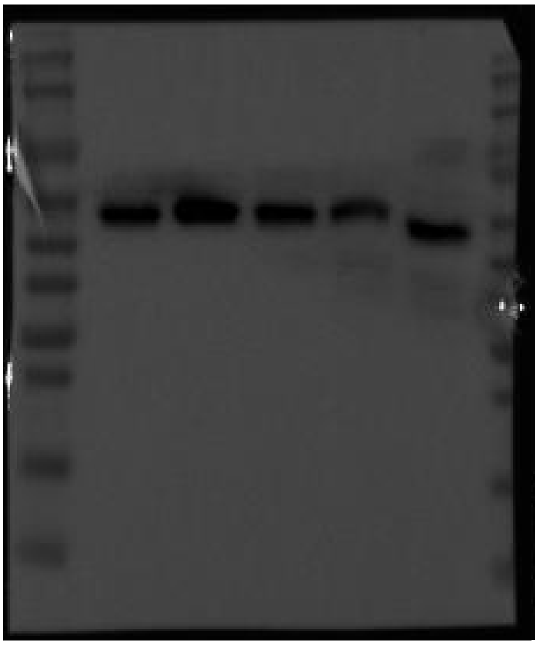

GAPDH

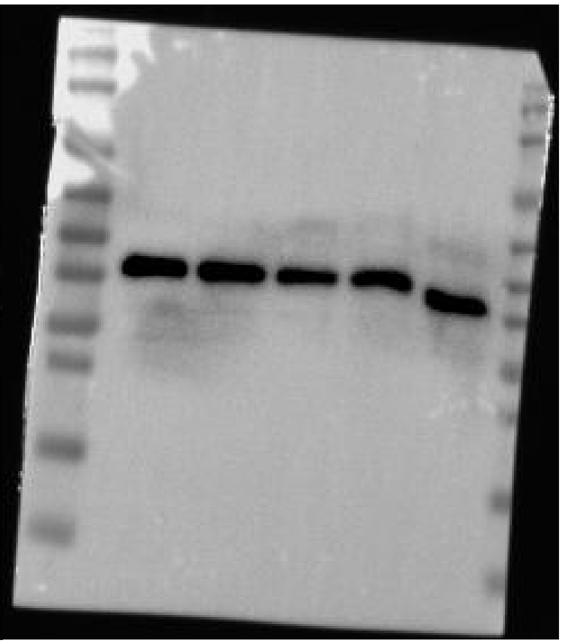

MMP13

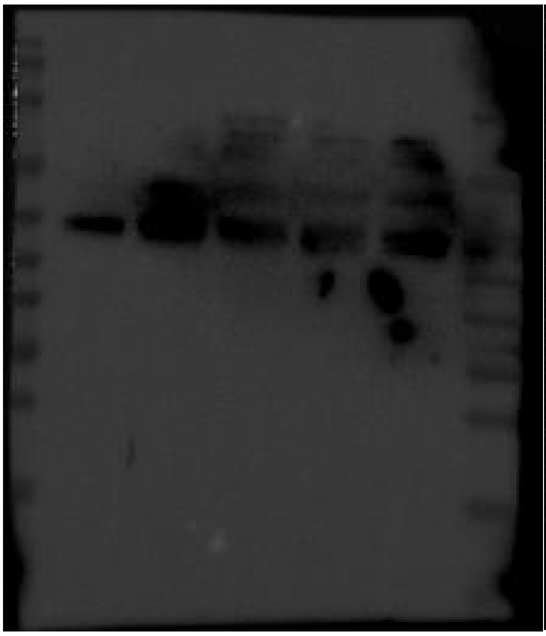

GAPDH

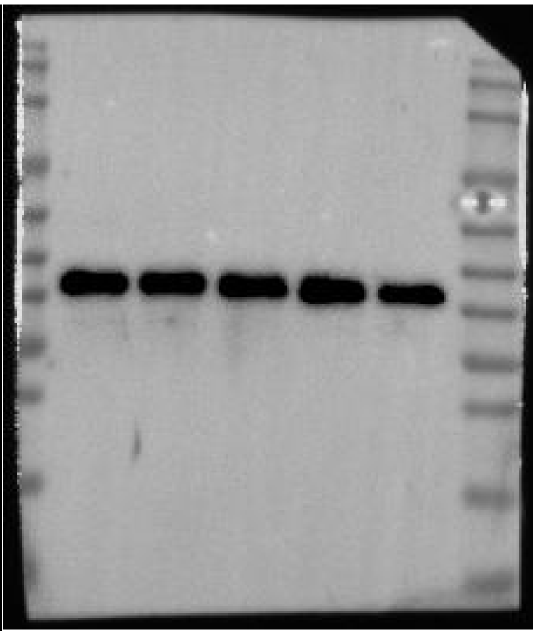

MMP13

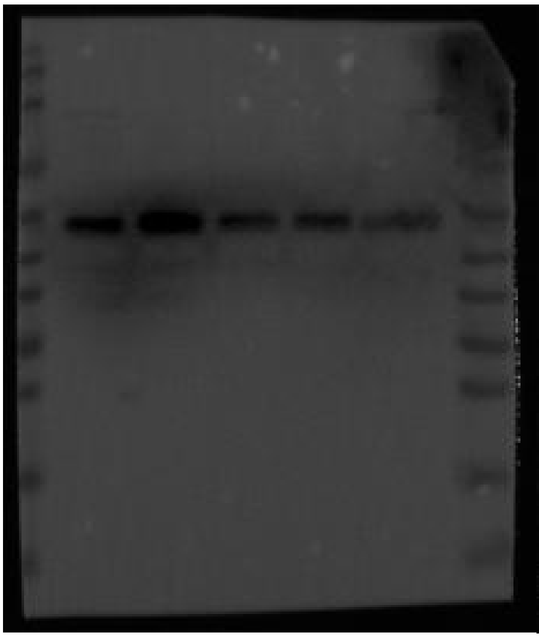

GAPDH

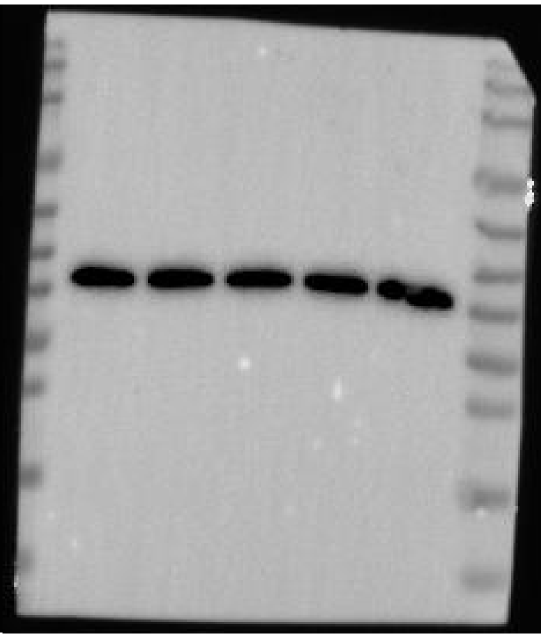

MMP13

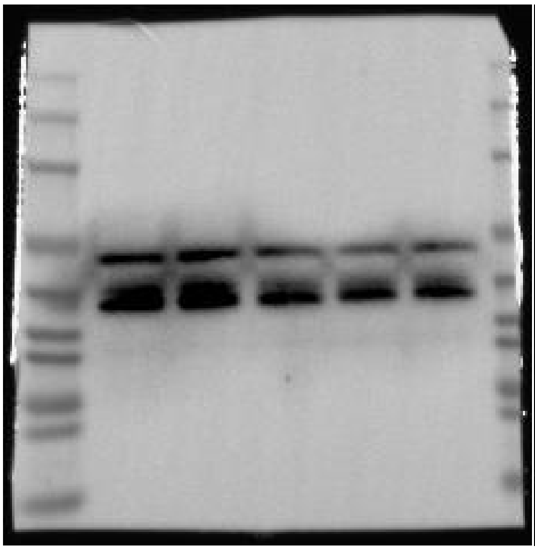

GAPDH

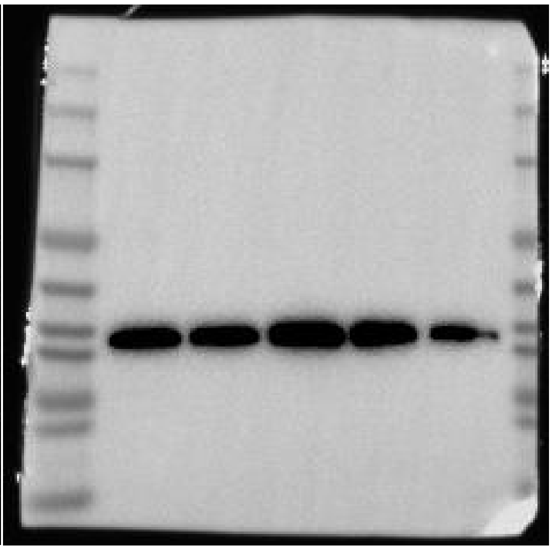

MMP13

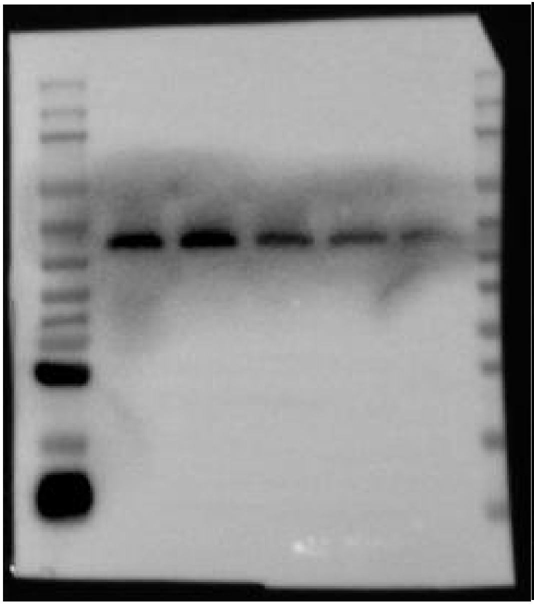

GAPDH

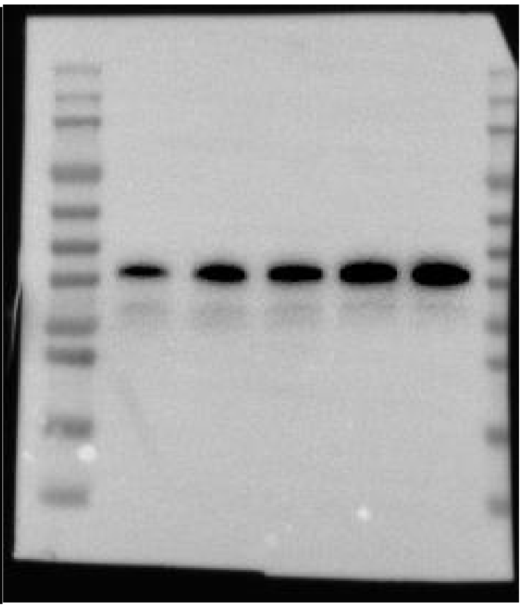

MMP13

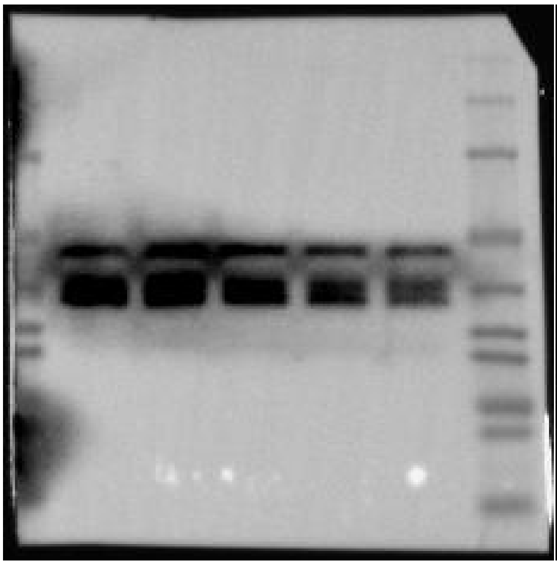

GAPDH

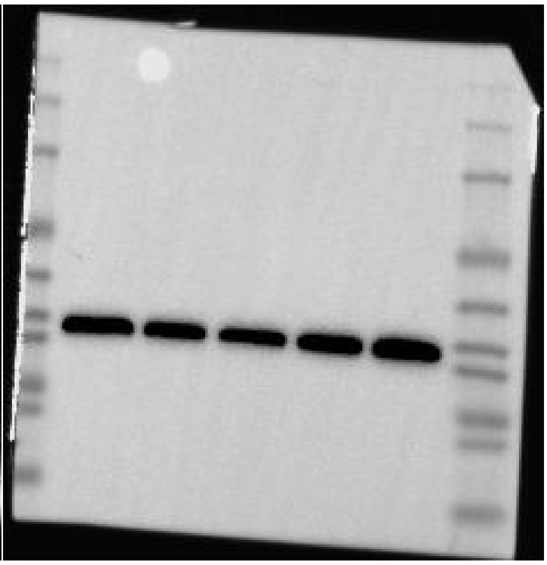

MMP13

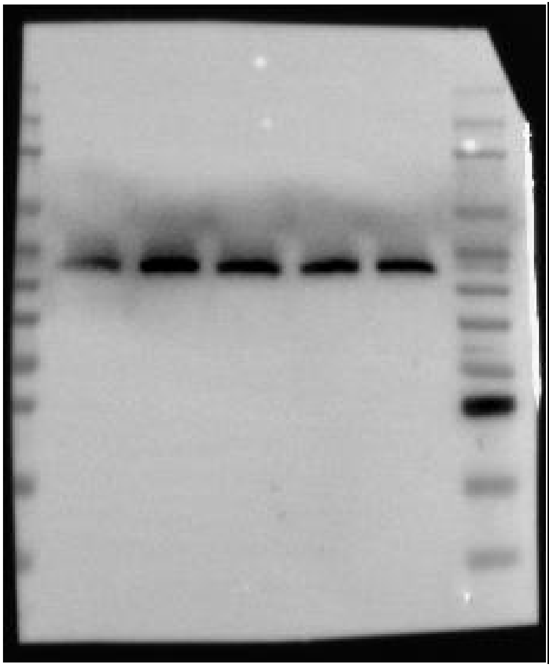

GAPDH

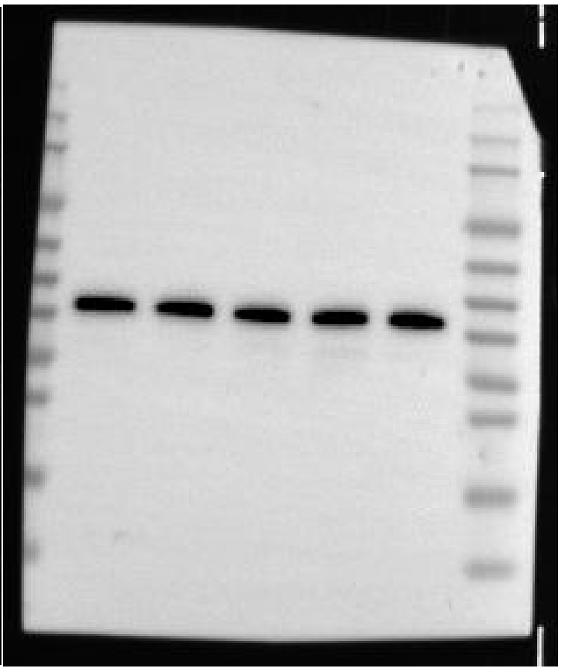

Figure 3B

ACAN

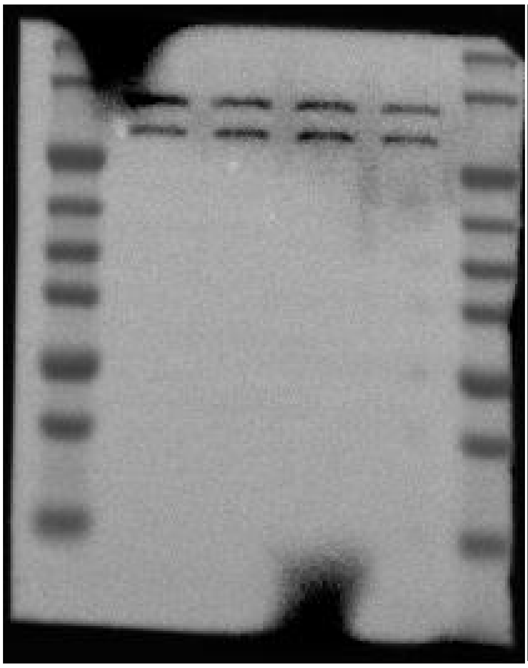

β-actin

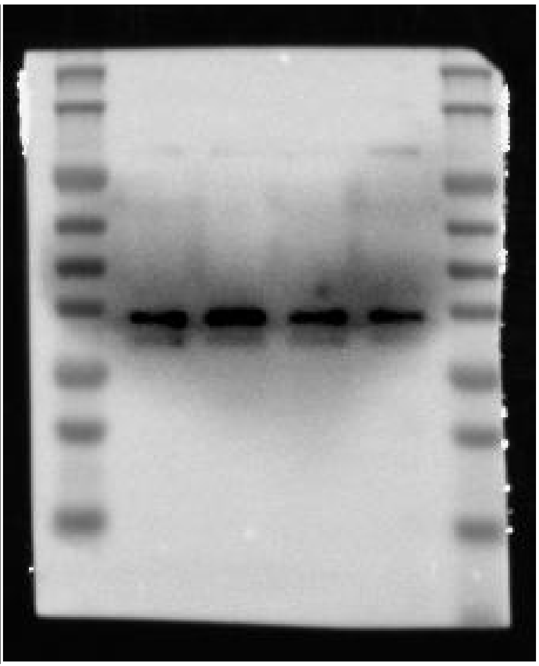

ACAN

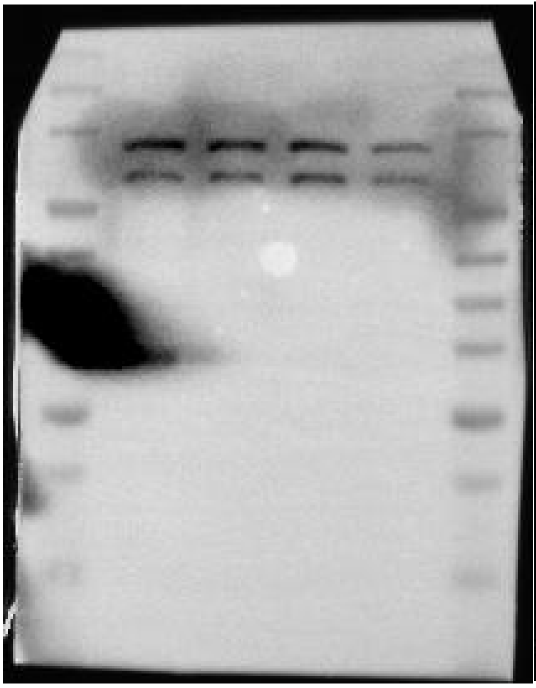

β-actin

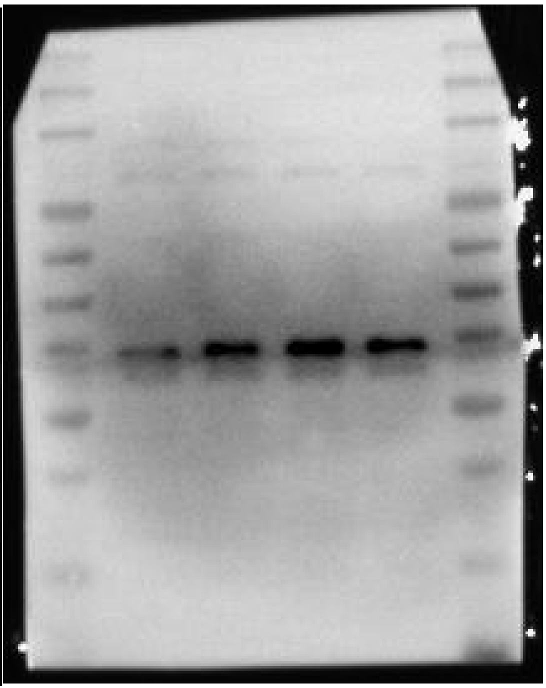

ACAN

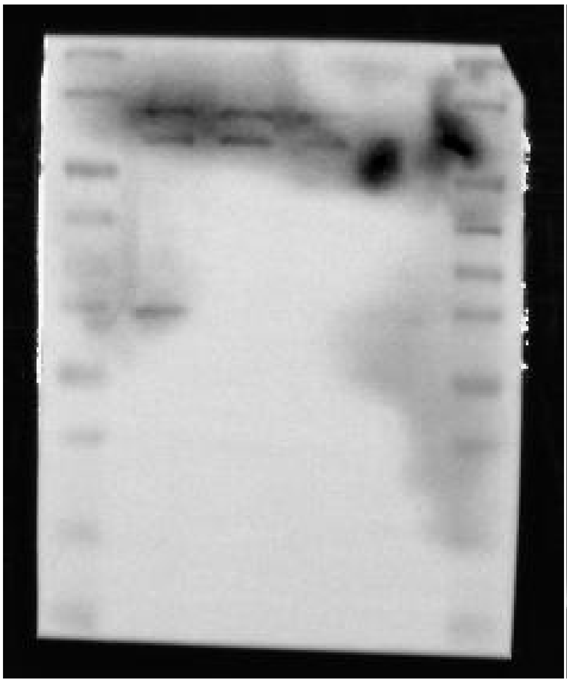

ACAN

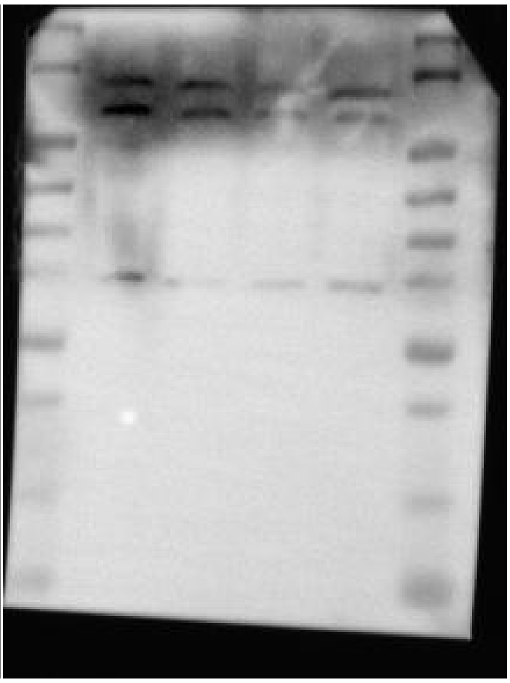

Caspase-1

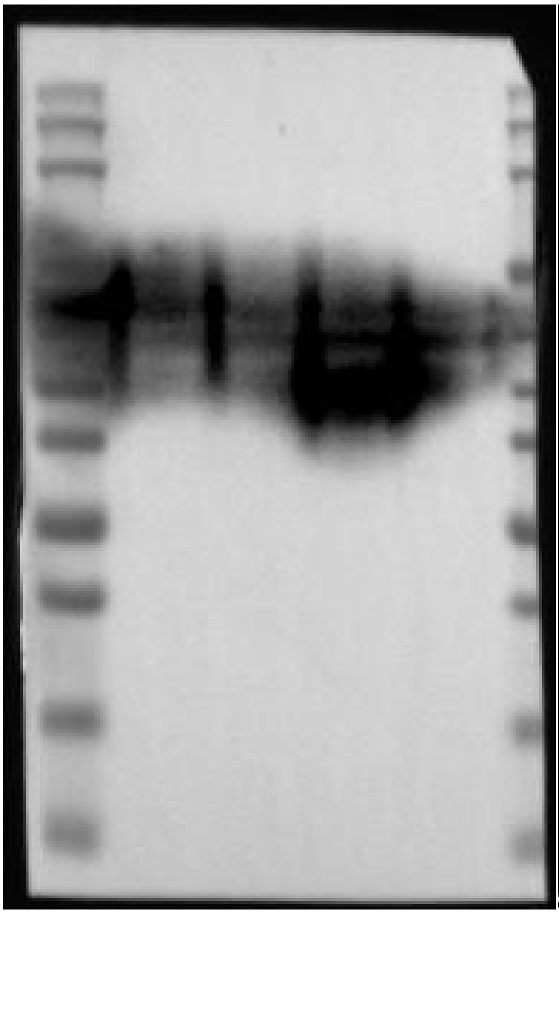

Caspase-1

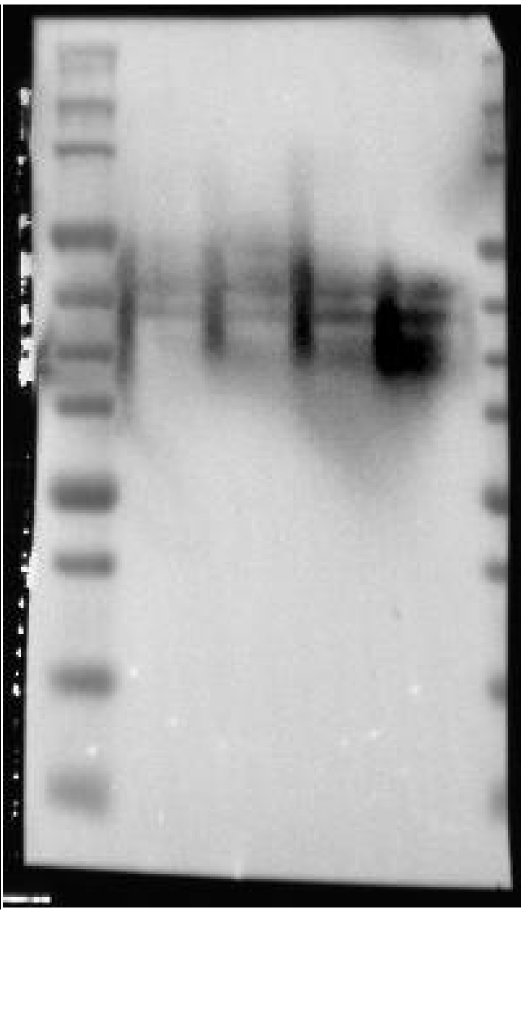

Caspase-1

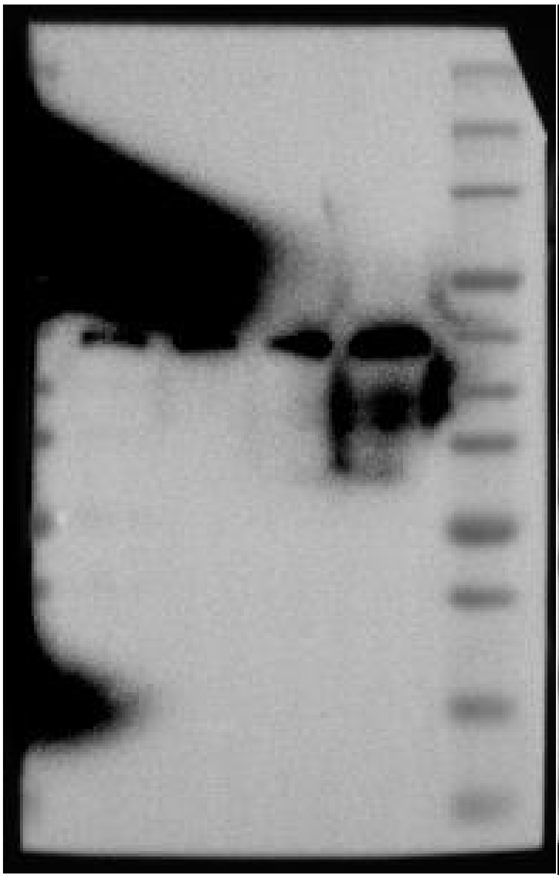

Caspase-1

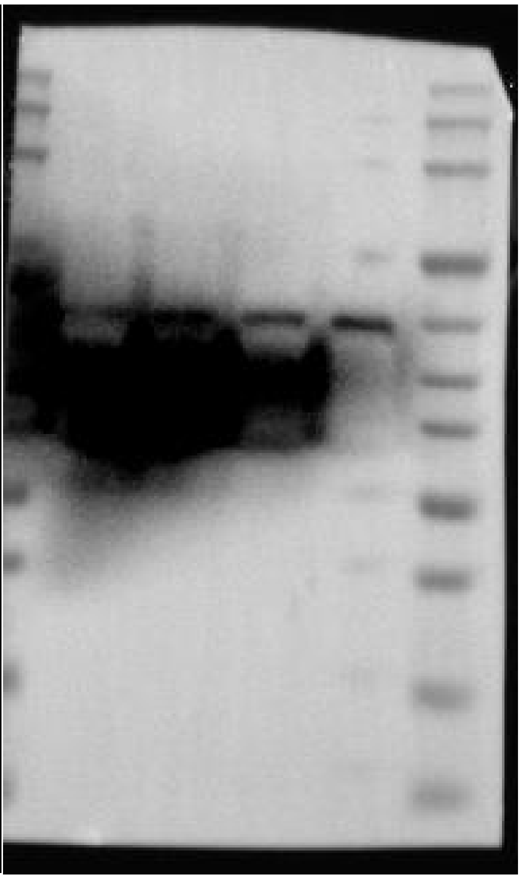

Caspase-1

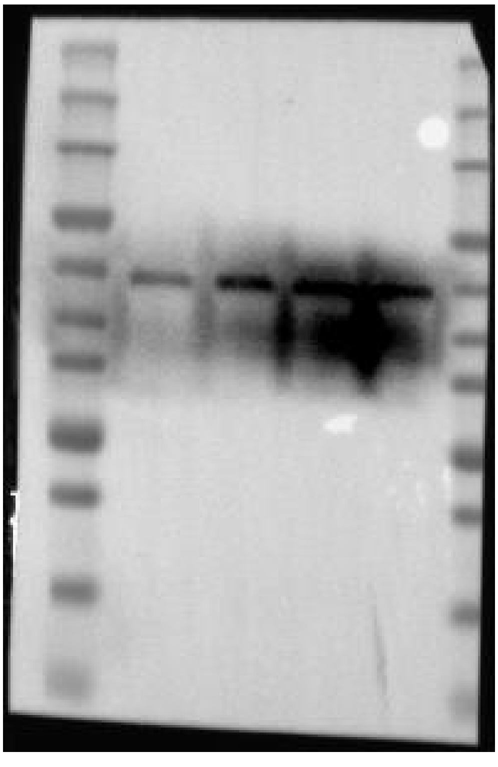

MMP13

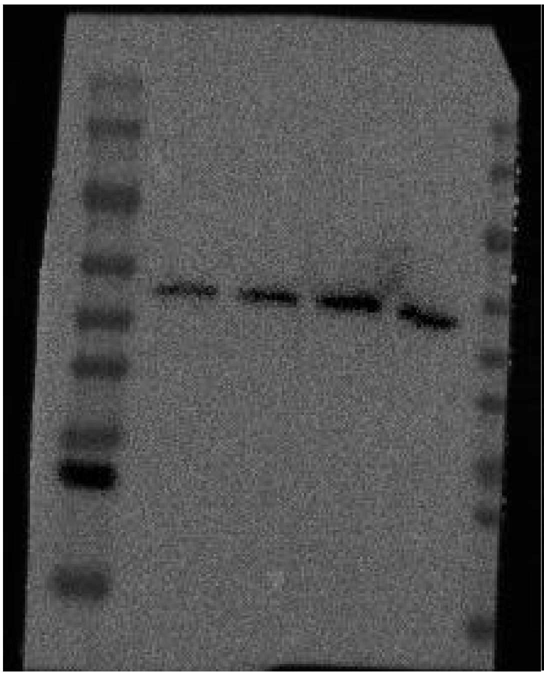

GAPDH

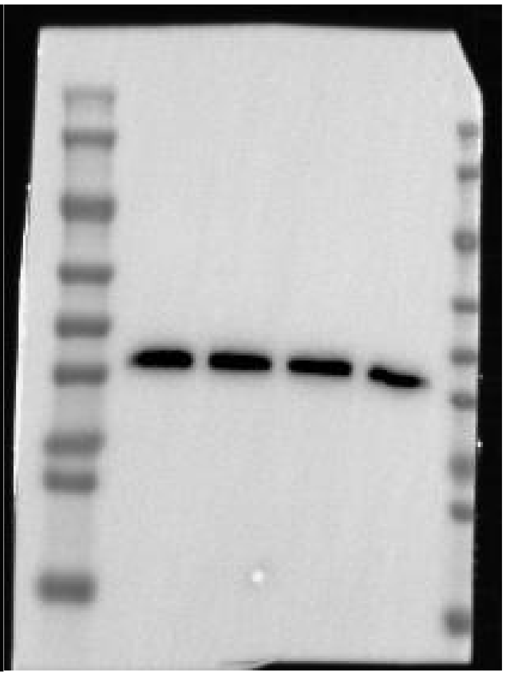

MMP13

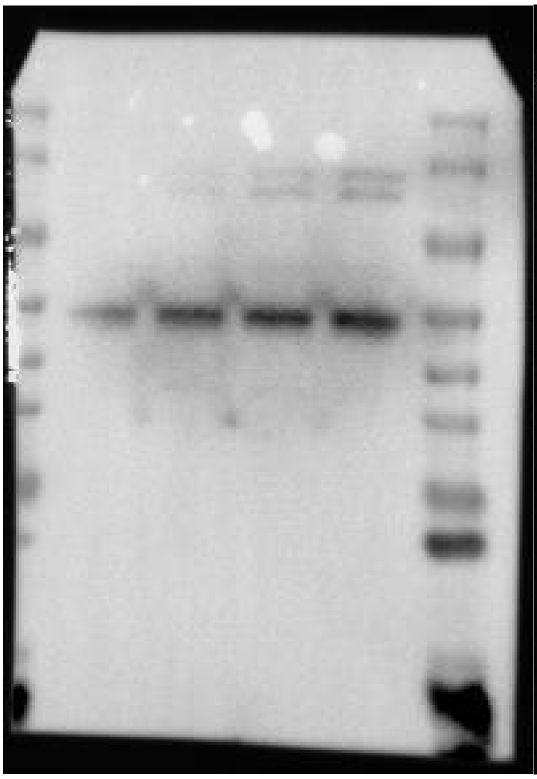

GAPDH

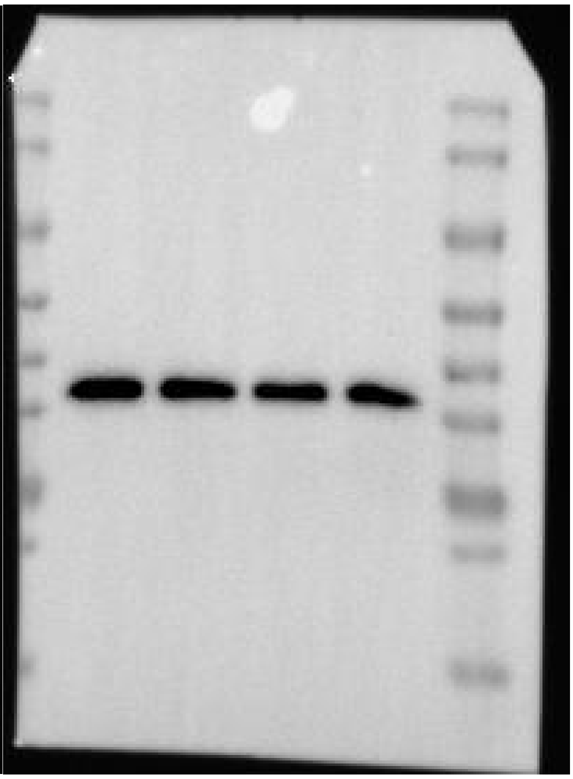

MMP13

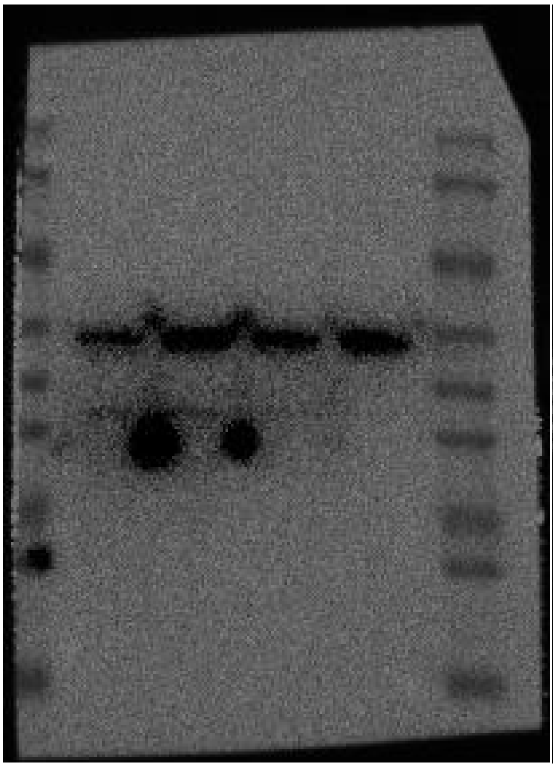

GAPDH

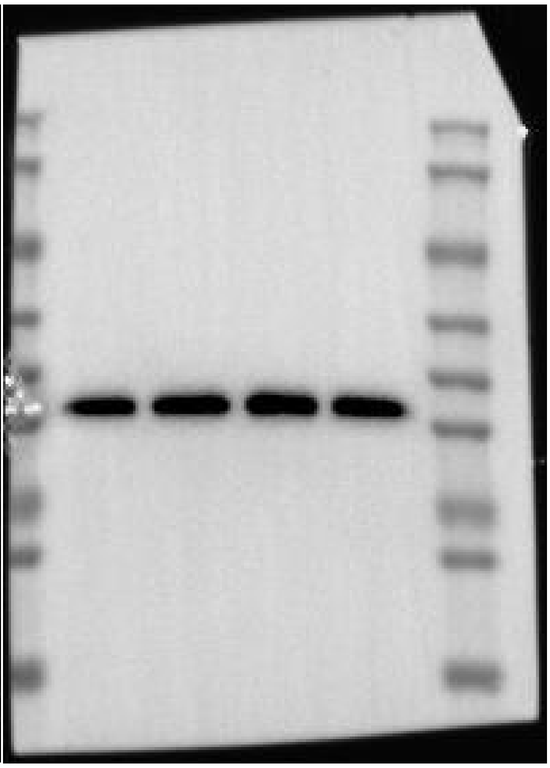

GAPDH

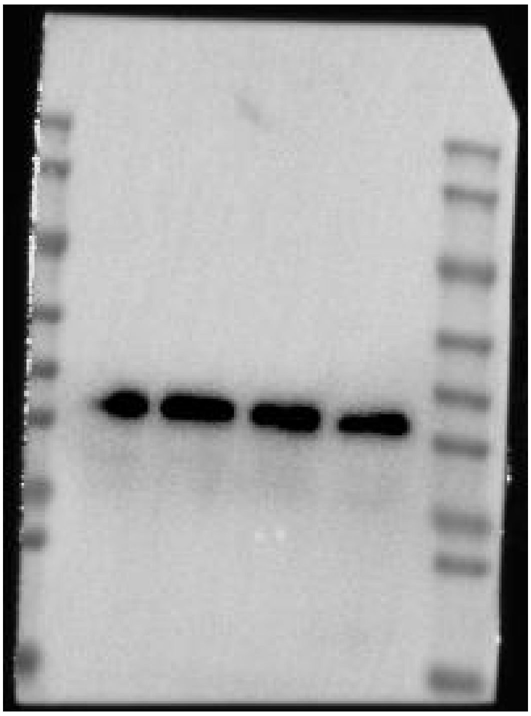

NLRP3

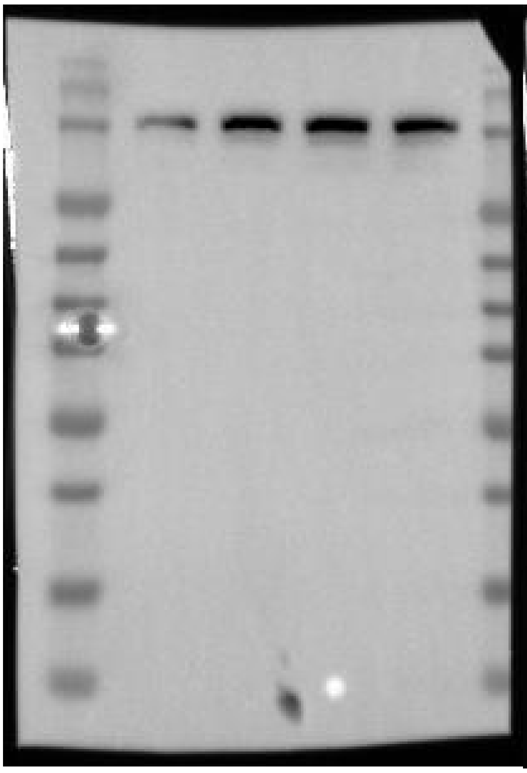

GAPDH

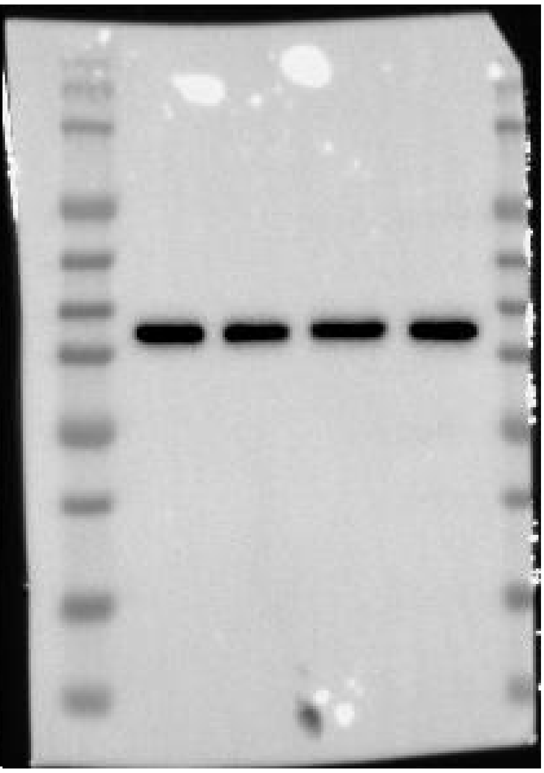

NLRP3

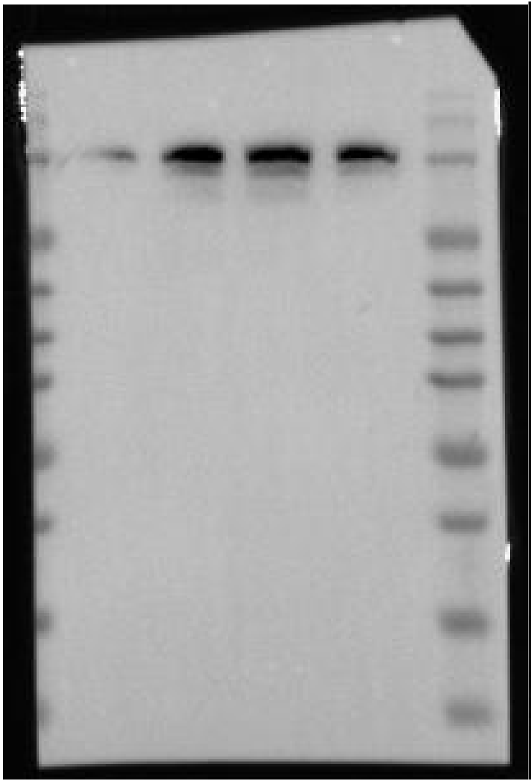

GAPDH

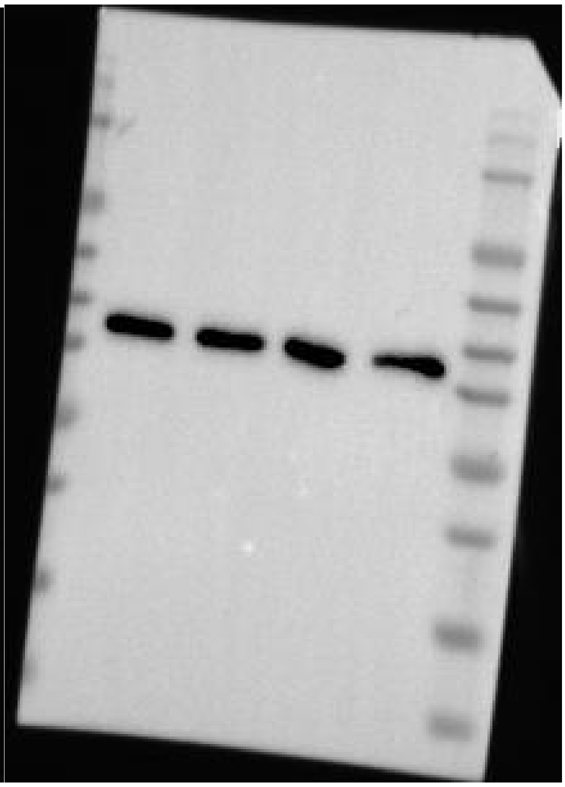

NLRP3

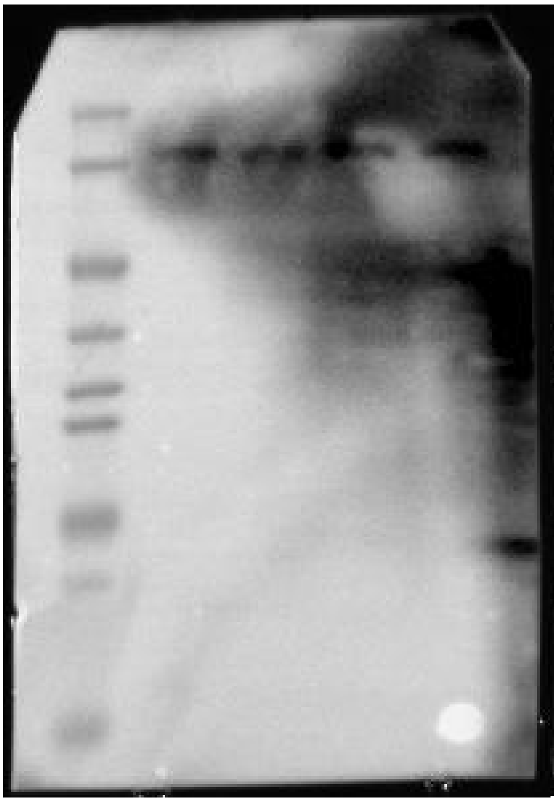

GAPDH

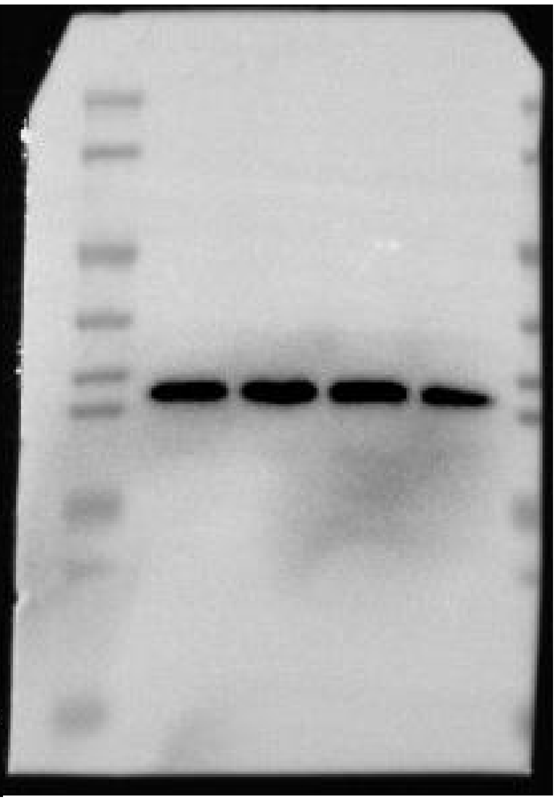

NLRP3

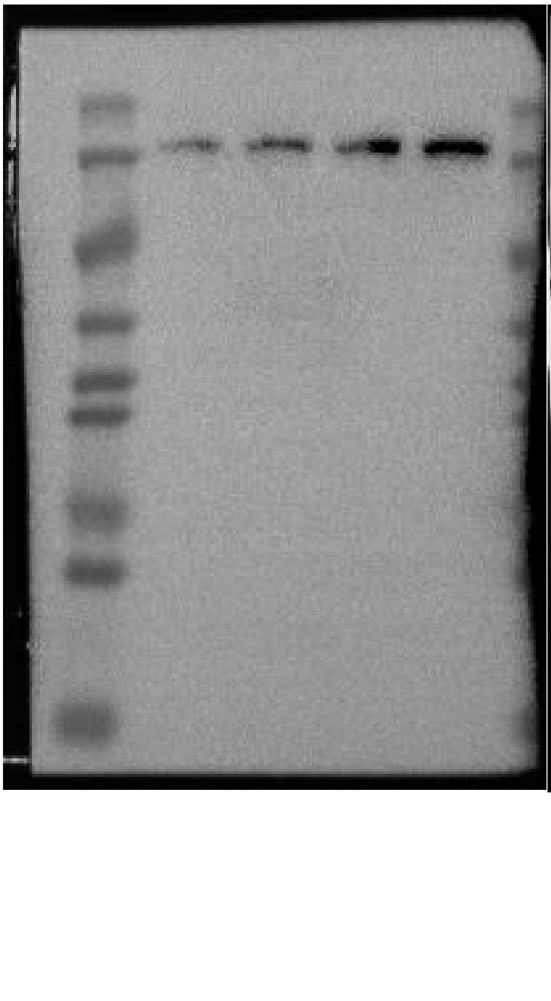

GAPDH

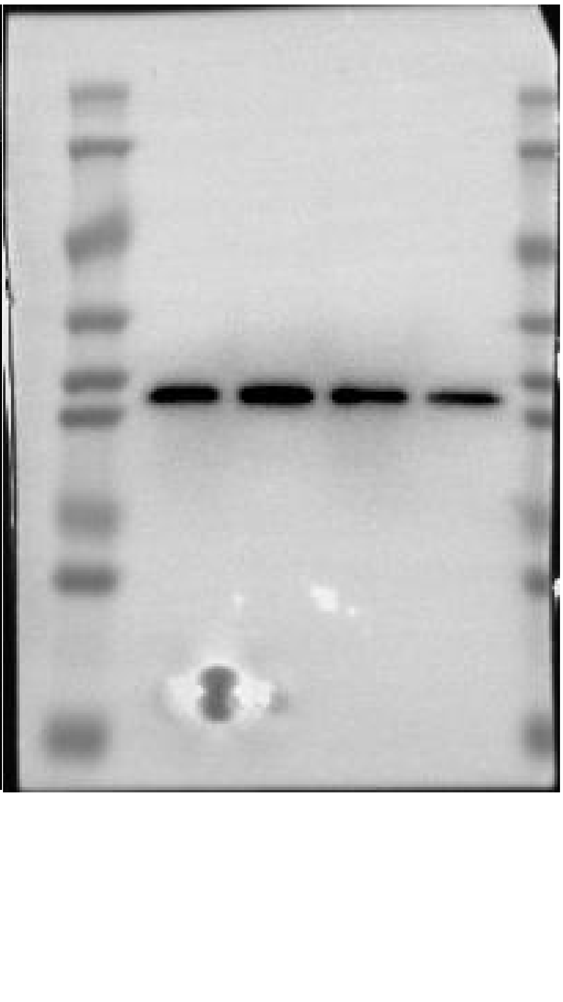

IL-18

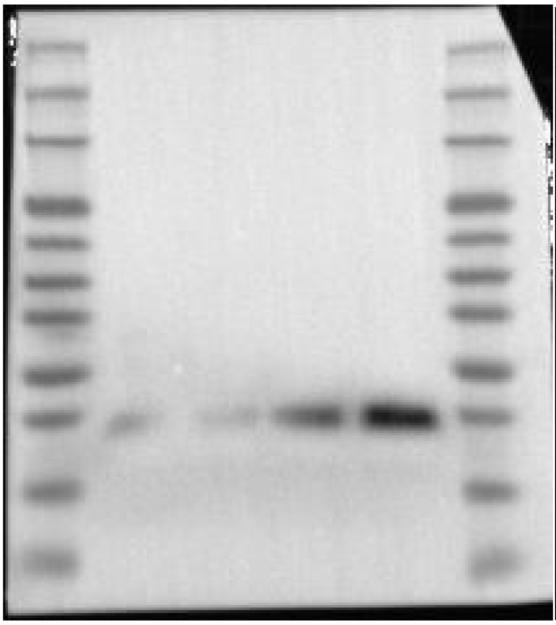

IL-18

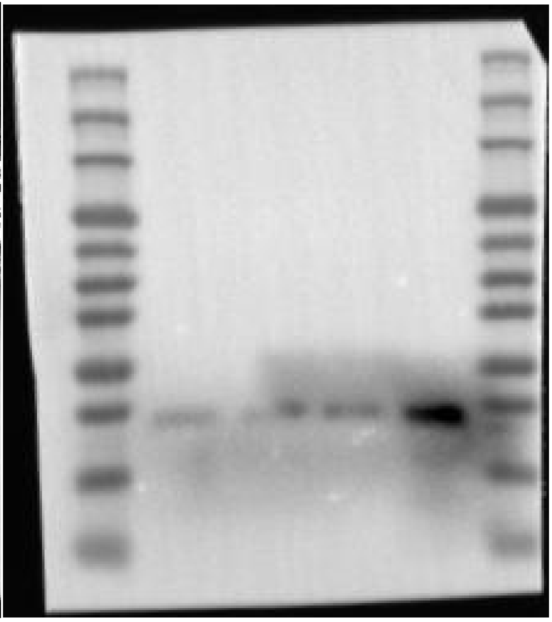

IL-18

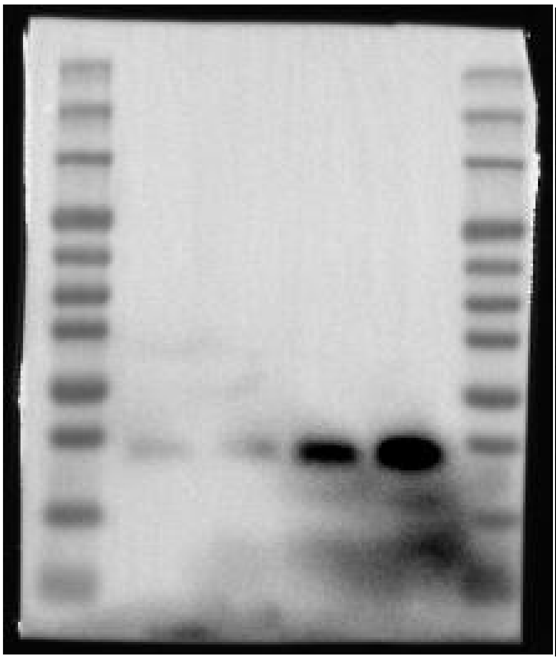

IL-18

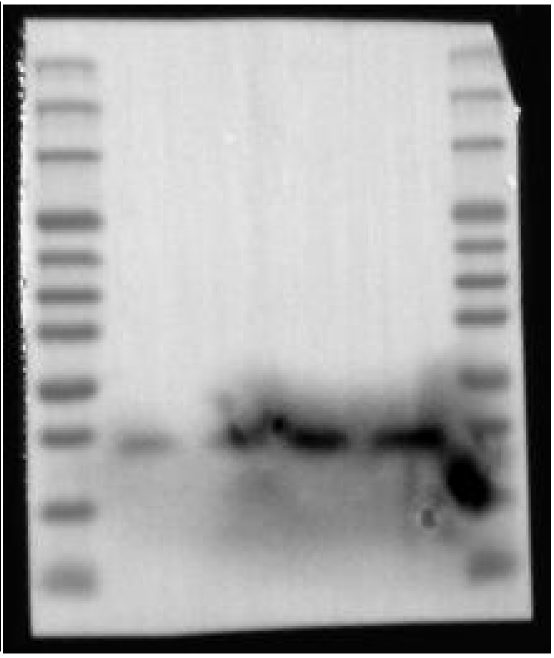

Supplement: Multimedia component 1 [file mmc1.pdf]
