# Supplementary material for: Mechanosensitive lncRNA H19 promotes chondrocyte autophagy, but not pyroptosis, by targeting miR-148a in post-traumatic osteoarthritis
Source: Noncoding RNA Res. 2024 Jul 31;10:163–76. doi: 10.1016/j.ncrna.2024.07.005 (PMC11470567; doi:10.1016/j.ncrna.2024.07.005)
Supplement: Multimedia component 2 [file mmc2.docx]

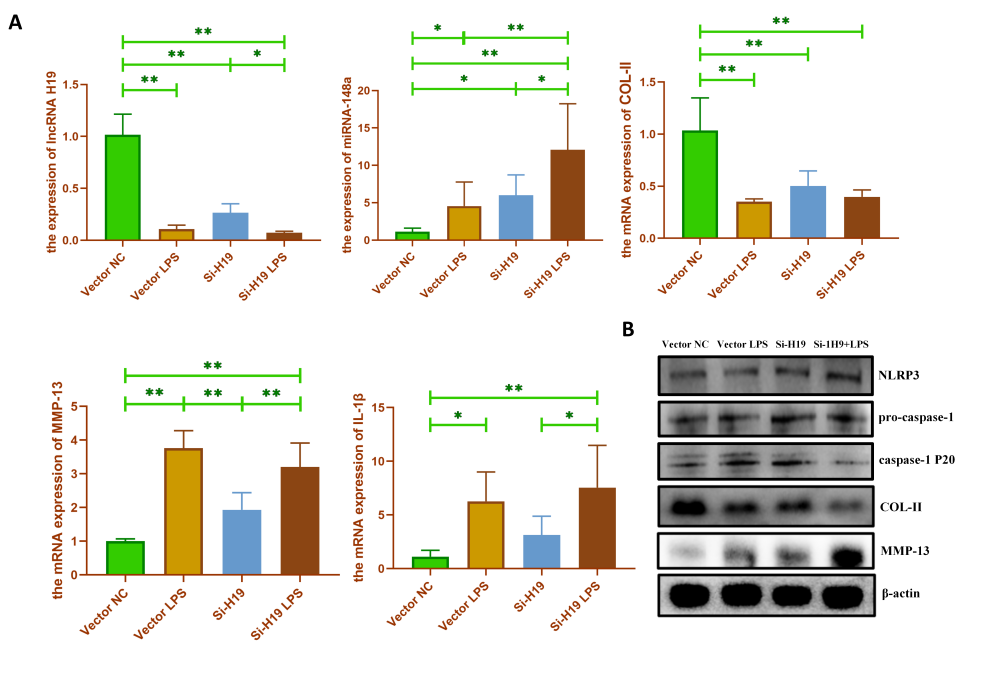


**Figure S1.** **Knockdown of lncRNA H19 had no significant effect on chondrocyte pyroptosis.** (**A**: RT-qPCR was used to detect the effect of lncRNA H19 knockdown on miR-148a and the mRNA expression of anabolism, catabolism, and inflammatory factors in LPS-induced chondrocytes. **B**: Western Blot was used to examine the effect of knockdown of lncRNA H19 on chondrocyte metabolism and pyroptosis-related protein markers in LPS-induced chondrocytes. Vector NC group: Vector negative control group; Vector LPS group: Vector + 10μg/ml LPS group; Si-H19 group: knockdown lncRNA H19 group; Si-H19 LPS group: knockdown lncRNA H19 + 10μg/ml LPS group, *P<0.05, **P<0.01)


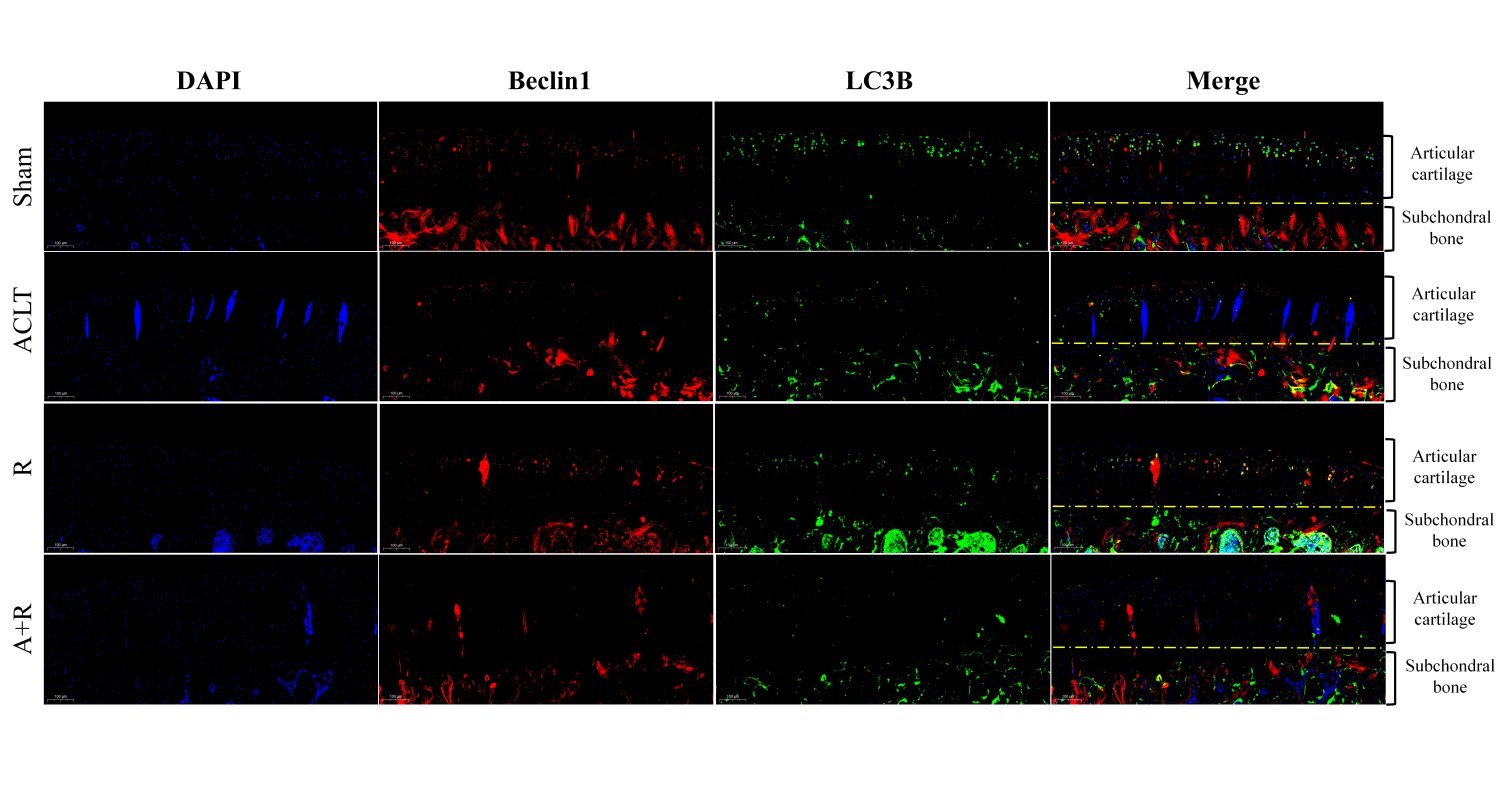


**Figure S2.** **Protein expression of autophagy-related indicators in rat articular cartilage by immunofluorescence.** (Sham group: Sham-operated group; ACLT group: anterior cruciate ligament transection surgery group; R group: treadmill running group; ACLT+R group: anterior cruciate ligament transection surgery plus treadmill running group)


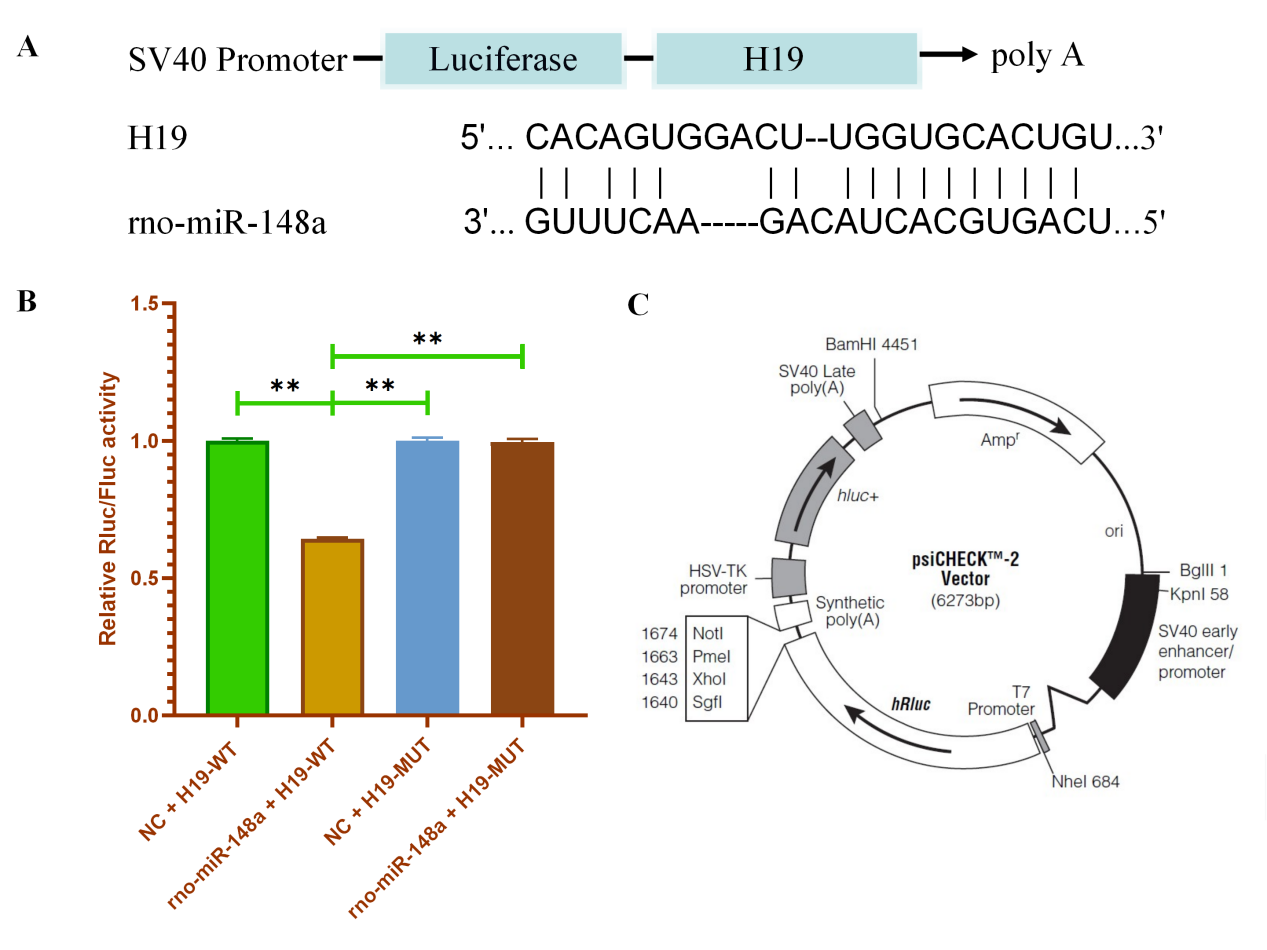


**Figure S3.** **The dual-luciferase reporter assay of miR-148a and lncRNA H19.** (**A**: The binding sites of rno-miR-148a and lncRNA H19. **B**: Expression changes of reporter genes in the H19-WT and H19-MUT groups. **C**: The psiCHECK-2 carrier profile.)
